# A data-driven simulation platform to predict cultivars’ performances under uncertain weather conditions

**DOI:** 10.1038/s41467-020-18480-y

**Published:** 2020-09-25

**Authors:** Gustavo de los Campos, Paulino Pérez-Rodríguez, Matthieu Bogard, David Gouache, José Crossa

**Affiliations:** 1 Departments of Epidemiology & Biostatistics and Statistics & Probability, IQ - Institute for Quantitative Health Science and Engineering, Michigan State University, East Lansing, MI USA; 2 Colegio de Postgraduados, CP 56230 Montecillos, Estado de México México; 3 Arvalis – Institut du Végétal, 6 Chemin de la côte vieille, 31450 Baziège, France; 4 Arvalis – Institut du Végétal, Station Expérimentale, 91720 Boigneville, France; 5 International Maize and Wheat Improvement Center (CIMMYT), Km 45, Carretera México-Veracruz, El Batán, Texcoco, Edo. de México México; 6 Present Address: Terres Inovia, 11 rue Gaspard Monge, 33600 Pessac, France

**Keywords:** Data integration, Agricultural genetics

## Abstract

In most crops, genetic and environmental factors interact in complex ways giving rise to substantial genotype-by-environment interactions (G×E). We propose that computer simulations leveraging field trial data, DNA sequences, and historical weather records can be used to tackle the longstanding problem of predicting cultivars’ future performances under largely uncertain weather conditions. We present a computer simulation platform that uses Monte Carlo methods to integrate uncertainty about future weather conditions and model parameters. We use extensive experimental wheat yield data (*n* = 25,841) to learn G×E patterns and validate, using left-trial-out cross-validation, the predictive performance of the model. Subsequently, we use the fitted model to generate circa 143 million grain yield data points for 28 wheat genotypes in 16 locations in France, over 16 years of historical weather records. The phenotypes generated by the simulation platform have multiple downstream uses; we illustrate this by predicting the distribution of expected yield at 448 cultivar-location combinations and performing means-stability analyses.

## Abstract (web summary)

Predicting crop performance in environments with limited field testing is challenging. Here the authors combine field experimental, DNA sequence, and weather data to predict genotypes’ future performance. They demonstrate the potential of this approach on a large dataset of wheat grain yield.

## Introduction

According to a recent report by the World Bank, overall food demand will increase by more than 50% by 2050^1^. This remarkable increase in food demand places enormous pressures on crop production. The same World Bank report concludes that crop yields will need to grow faster than historically to meet the anticipated food demand. The genetic improvement of crops is one of the main ways in which modern agriculture can maintain and increase production levels while reducing its environmental impacts.

In plants, genetic and environmental factors can interact in complex ways giving rise to substantial genetic-by-environment (G×E) interactions^2^. This source of variation can be used to select genotypes adapted to specific environments^3^. However, making selection decisions and agronomic recommendations is exceptionally challenging because future environmental conditions are mainly uncertain. Indeed, accurate predictions of future performances in target environments require considering the possible weather conditions that may occur within a region and how individual genotypes are expected to react to those conditions. Extensive field testing, including evaluating genotypes over many years and across multiple locations, is required to make such predictions. However, efficient trial networks can only test genotypes over a limited number of years and testing sites. Thus, in the early stages of their breeding cycle, genotypes are often advanced without being tested under weather conditions that may critically affect their performance (e.g., cold, heat, or drought stress).

**Fig. 1:**
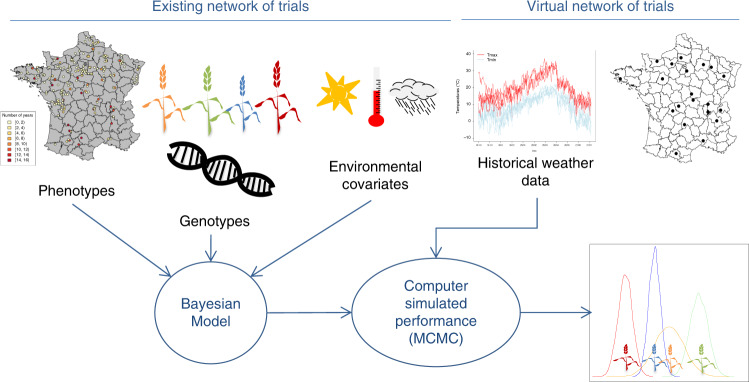
Computer-simulated performance of candidate genotypes in target locations. The proposed computer-simulation platform uses phenotype, genotype (e.g., SNPs), and environmental covariate data collected in an existing network of trials and historical weather records at target locations (“virtual network of trials”) to simulate the performances of selected genotypes in target locations, under possible weather conditions.

We propose that computer simulations that integrate field trial data, DNA sequences, and historical weather records can be used to address the difficult task of predicting genotype performance and stability using limited years of field testing per genotype. Figure 1 summarizes the proposed simulation framework. Our approach builds on modern genomic models that integrate DNA sequences (e.g., single nucleotide polymorphisms—SNPs) and environmental covariates (ECs^4,5^). The use of ECs as a means to characterize the environmental conditions that occurred during a growing season enables us to link past field trial data with historical (or simulated) weather records that describe environmental conditions that are likely to occur in a location or region.

Our approach is largely data-driven; we use a G×E model incorporating SNPs and ECs to learn how each cultivar reacted to the environmental conditions. We then use these patterns, together with DNA polymorphisms and historical weather records, to simulate the expected performance of specific genotypes at specific locations. The Monte Carlo (MC) method used to simulate phenotypes integrates uncertainty about future weather conditions and model parameters (e.g., SNP or EC effects and their interactions).

We apply the proposed simulation platform to wheat data from an extensive trial network generated by Arvalis-Institut-du-végétal (Arvalis). The data set comprises (*n* = 25,841) wheat grain yield records from French-registered cultivars, linked to DNA sequences (167,440 SNPs) and (106) ECs describing temperature, radiation, and water availability in different phases of the crop cycle. We use this extensive data set to fit the models proposed by Jarquín et al.^4^ with various genetic and environmental specifications. We validate the predictive performance of the models using a “leave-trial-out” cross-validation. Subsequently, we use samples from the posterior distribution of the model parameters and ECs derived from historical weather records to simulate the performance of 28 wheat lines across possible occurrences of environmental conditions at 16 target locations representative of major French wheat-producing regions. Finally, we use the simulated data for two downstream analyses: prediction of expected grain yield (and its distribution) at target cultivar–location combinations, and mean and stability analyses, based on Finlay–Wilkinson (FW) regression^6^. Our results show that wheat yield forecasts of individual varieties at target locations derived from simulations integrating many years of historical weather data are more precise than forecasts that can be derived from trial data alone.

## Results

### An extensive, highly-connected trial network

**Fig. 2:**
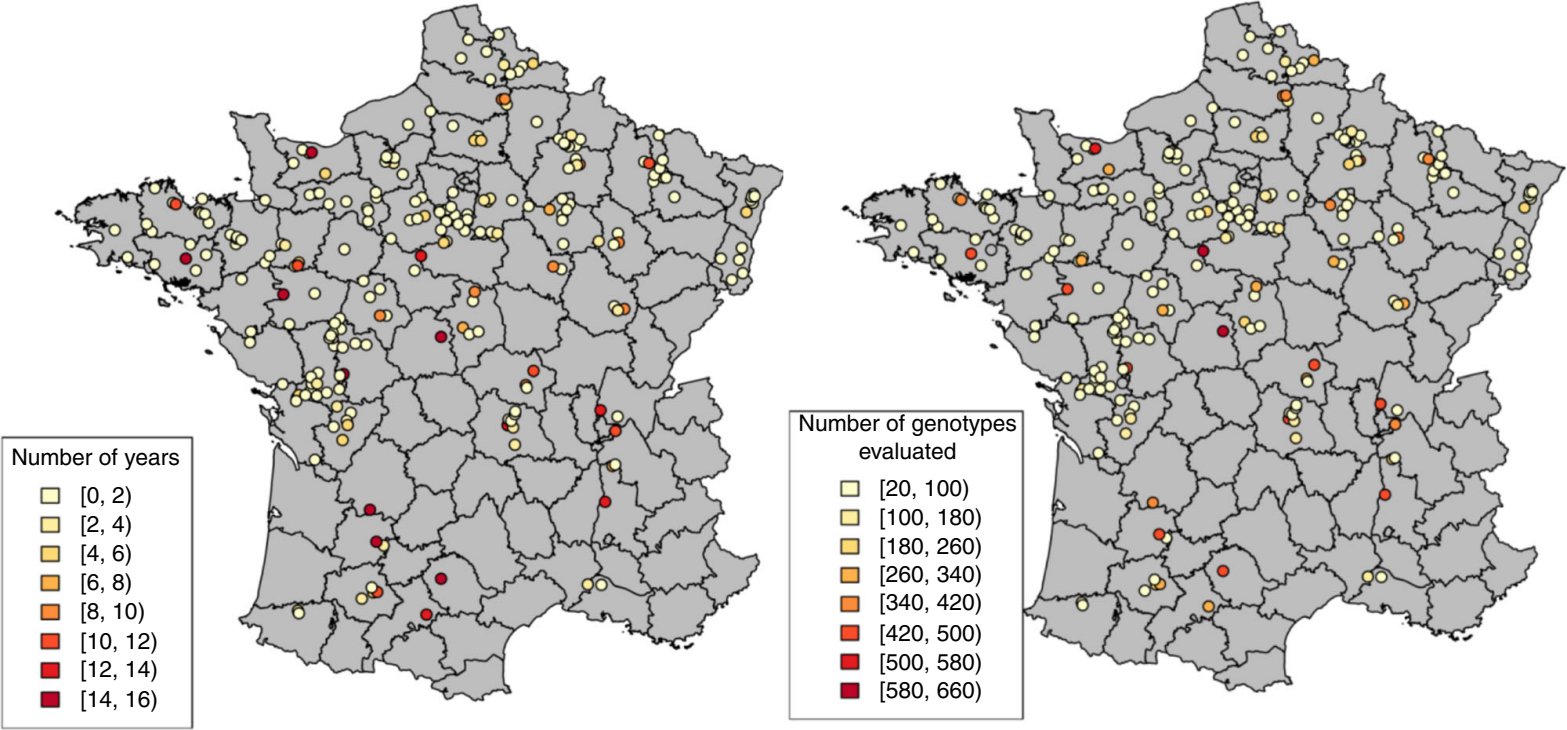
Network of trials. Each dot represents a trial location; the color scale is used to denote the number of years with records for each location (left) and the number of genotypes tested at each location (right). (Clustering based on minimum temperature, maximum temperature, rain, and total wheat area is shown in Supplementary Fig. 4).

Field trial data were generated by Arvalis (https://www.arvalisinstitutduvegetal.fr) and included 25,841 grain yield records collected from 1998 to 2014 at 242 locations (Fig. 2). There were 752 year–location combinations with one (un-replicated) trial per year–location. In total, 481 French-registered cultivars were tested. All trials were connected through a common tester and by many other cultivars that generated partial connections between trials. On average, each trial was connected with at least 340 other experiments through at least five genotypes (see “Methods” and Supplementary Fig. 1 for further details). All testing locations had a meteorological station within 10 km from where environmental data were retrieved. All trials received fungicide and seed treatments.

The average grain yield (standardized at 15% moisture) was 9.49 (±1.50) tons per hectare. The distribution of grain yield was reasonably symmetric (Supplementary Fig. 2).

Each variety was genotyped with an Axiom high-density genotypic platform (Affymetrix Inc., Santa Clara, CA) containing 420K SNPs^7^; these genotypes were generated within the Breedwheat project (ANR-10-BTR-03). After standard quality control, we had a total of 167,440 SNPs available for analyses. Principal components derived from SNP genotypes revealed a weak substructure among genotypes (Supplementary Fig. 3).

Environmental data consisted of 106 environmental covariates (EC) generated using a crop model developed by Arvalis^8^. The crop model computes EC values based on predicted growth stages and weather records (temperature, radiation, rainfall). The output of the model consists of ECs describing critical temperatures, radiation, and water availability for eight distinct phases of crop phenology. A complete list of the ECs used is presented in Supplementary Table 1. Further details about how the ECs were derived are provided in the “Methods” section and in Soenen et al.^8^.

The ECs used in this study were used before to characterize French wheat-growing environments^9^ and to predict regional wheat yield in France^10^. A principal component analysis of the 106 ECs revealed almost no structure (Supplementary Fig. 3). To validate the ECs, we regressed the trial means on the 106 ECs and quantified the ability of the ECs to predict trial means in cross-validation (see “Methods” for more details). Over 100 training-testing partitions, the average testing correlation between the EC-predicted means and the (BLUE of the) trial means was 0.600 (+/−0.04).

### EC captured 50% the environmental variance

We used a sequence of models to evaluate the proportion of grain yield variance explained by genetic and environmental factors (Table 1), and to reveal the fraction of those variance components that could be captured using SNPs and ECs.

**Table 1:** Estimated variance components [95% credible interval] for wheat grain yield.

Model	Year	Location	Y×L	Cultivar	Env. Cov.	SNP×EC	Error
TL (baseline)	0.289	0.780	0.944	0.191	–	–	0.215
	[0.210,0.373]	[0.640,0.927]	[0.878,1.018]	[0.184,0.199]	–	–	[0.214,0.215]
GW	–	–	–	0.158	0.892	–	1.300
	–	–	–	[0.146,0.172]	[0.863,0.922]	–	[1.296,1.304]
GW-G×W	–	–	–	0.160	0.863	0.074	1.2417
	–	–	–	[0.145,0.177]	[0.830,0.897]	[0.062,0.086]	[1.234,1.250]
TGW	0.196	0.495	0.935	0.190	0.154	–	0.212
	[0.126,0.280]	[0.360,0.627]	[0.867,1.012]	[0.181,0.198]	[0.105,0.213]	–	[0.211,0.213]
TGW-G×W (full model)	0.175	0.525	0.916	0.184	0.140	0.054	0.170
	[0.110,0.248]	[0.382,0.629]	[0.848,0.992]	[0.173,0.195]	[0.090,0.198]	[0.050,0.058]	[0.168,0.172]

*T* trial, *Y* year, *L* location, *Y×L* year-by-location, *G* SNP genotypes, *W* environmental covariates, *G×W* SNPs-by-Env. Cov.

The baseline model (TL) included an intercept plus the random effects of year (*Y*_*i*_), location (*L*_*j*_), year–location (*YL*_*ij*_), and cultivar (*V*_*k*_); thus

1 $${\mathrm{TL{:}}}\;y_{ijk} = \mu + Y_i + L_j + YL_{ij} + V_k + \varepsilon _{ijk},$$

where $Y_i\sim {\mathrm{NIID}}(0,\sigma _Y^2)$, $L_j\sim {\mathrm{NIID}}(0,\sigma _L^2)$, $YL_{ij}\sim {\mathrm{NIID}}(0,\sigma _{YL}^2)$, $V_k\sim {\mathrm{NIID}}(0,\sigma _V^2)$, and $\varepsilon _{ijk}\sim {\mathrm{NIID}}\left( {0,\sigma _\varepsilon ^2} \right)$; this model does not incorporate any SNP or EC data.

Estimates indicate that environmental differences between trials (i.e., those due to year, location and year-by-location effects) explained 83% of the grain yield variance [(0.289 + 0.780 + 0.944)/(0.289 + 0.780 + 0.944 + 0.191 + 0.215), Table 1]. Approximately one-half of the between-trial variance corresponds to year-by-location interactions [0.944/(0.289 + 0.780 + 0.944)], thus highlighting the importance of accounting for year-to-year variations in environmental conditions. In the baseline model, the amount of variance explained by the main effect of the genotypes was ~8% of the total variance and about 50% of the within-trial variance.

We expanded the baseline model by adding the cultivar-by-year (V × Y) and cultivar-by-location interactions (V×L) (we did not include cultivar-by-year location because in the trials, there was only one plot per genotype–year–location). The interactions captured a very small amount of variance (0.0323 +/− 0.0019 and 0.0415 +/− 0.0023, for V×L and V×Y, respectively). Of the two terms, only the cultivar-by-location term could be learned from past data. However, the amount of variance captured by this term is small, and accurate prediction of V×L would require evaluating cultivars over many years at the same location, which is both costly and inefficient. Therefore, instead of leveraging V×L and V×Y for prediction, we focused on modeling those effects using SNPs, ECs, and their interactions.

To assess the proportion of genetic variance that can be captured by SNPs, we modeled the cultivar effect (*V*_*k*_) using assumptions of the GBLUP model^11^. Thus, ${\boldsymbol{V}} = \left\{ {V_k} \right\}\sim N\left( {{\mathbf{0}},{\boldsymbol{G}}\sigma _V^2} \right)$, where ***G*** is an (SNP-derived) additive relationship matrix, and $\sigma _V^2$ is a genomic variance. Likewise, we used an “EBLUP” model to introduce the effects of the ECs; thus ${\boldsymbol{w}} = \{ {w_{ijk}} \}\sim N\left( {{\mathbf{0}},{\mathbf{\Omega}}\sigma _{EC}^2} \right)$ where ***w*** is a vector of year-location-cultivar effects and **Ω** is an environmental similarity matrix derived from the ECs (see “Methods” for more details). Therefore, the GW model specifies

2 $${\mathrm{GW{:}}}\;y_{ijk} = \mu + V_k + w_{ijk} + \varepsilon _{ijk}$$

In the GW model, the genomic term (*V*_*k*_) captured ~83% of the variance of the genotypes (0.158/0.191); however, the ECs captured ~44% of the between-trial variance [0.892/(0.289 + 0.780 + 0.944)]. We conclude that there was almost no “missing heritability”^12^ and that there was a sizable (~56%) “missing environmentability”. The latest could be attributed to deficiencies in the ECs (e.g., lack of data on soil fertility) and to limitations in the model used to link ECs with grain yield (e.g., absence of non-linear effects or interactions between ECs).

Next, we added interactions between SNPs and ECs to the GW model (GW-G×W). With the number of SNPs and ECs involved in this study, modeling all possible pairwise SNP-by-EC interactions is computationally very challenging. However, SNP-by-EC interactions can be modeled (implicitly) using a Gaussian random effect with a covariance structure which is the Hadamard product (i.e., cell by cell) of the genomic (***G***) and environmental (**Ω**) covariance structures^4^. Thus, to introduce SNP-by-EC interactions we used the following model

3 $${\mathrm{GW}} - {\mathrm{G}}\times{\mathrm{W}{:}}\;y_{ijk} = \mu + V_k + w_{ijk} + VW_{ijk} + \varepsilon _{ijk},$$

where *μ*, *V*_*k*_, and *w*_*ijk*_ are as in Eq. (2), and *VW*_*ijk*_ is a Gaussian random effect representing SNP-by-EC interactions that has a zero mean and (co)variance function proportional to the product of the genomic (*G*_*ii*′_) and environmental $({\Omega} _{ijk,i\prime j\prime k\prime })$ similarity between entries, ${\mathrm{Cov}}\left( {VW_{ijk},VW_{i\prime j\prime k\prime }} \right) \propto G_{ii\prime}\,{\mathrm{{\Omega} }}_{ijk,i\prime j\prime k\prime }$ (see “Methods” for further details).

In the GW-G×W model, the regression on SNPs captured ~83% of the variance associated with genotypes (0.16/0.191), while the ECs captured ~42% of trial variance [0.863/(0.289 + 0.780 + 0.944)]. Relative to the additive effects of model GW, adding the interactions between SNPs and ECs led to a reduction in the error variance of about ~5% (0.074/1.300). However, the error variance of the GW-G×W model was substantially larger than the error variance of our baseline model, reflecting that even after including SNP-by-EC interactions, the model did not fully capture environmental effects.

Therefore, we then combined the trial information (year, location, and year-by-location) with SNPs and ECs. We did this without including SNP-by-EC interactions (model TGW = trials, genotypes and ECs, Eq. (4) in “Methods”) and including those interactions (TGW-G×W, see Eq. (5) in “Methods”). The full model (TGW-G×W) captured the same amount of variance as the baseline model, with (almost) all of the genetic variance being captured by SNPs and a sizable fraction of the environmental variance captured by ECs.

### Models achieved moderately high prediction accuracy

We used 10-fold cross-validation (CV), with trials (i.e., entire year-location combinations) assigned to folds, to assess each of the models’ ability to predict grain yield in year-locations not used to train the model. For each fold, we fitted models using data from the remaining ninefold and used the fitted model to predict yield in the trials assigned to the left-out fold. This was repeated 10 times, and each time onefold was left aside for testing. The assignment of year-locations to folds poses a prediction problem similar to the one faced when one uses data from some year-locations to predict the performance of genotypes in other year-locations. We chose this approach because it represents the prediction problem that one faces when simulating genotypes’ performance in future years at locations present in the network of trials.

**Fig. 3:**
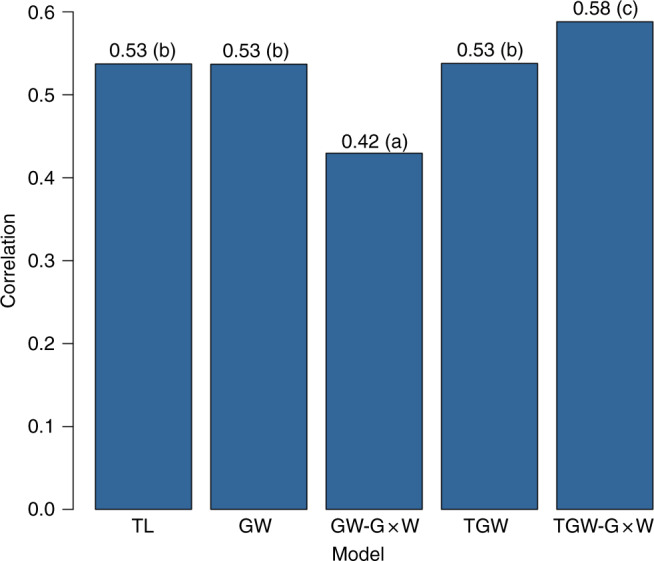
Average within-trial correlation between predicted and observed yield. Results were obtained with a ‘leave-trial-out’ cross-validation. Letters indicate differences at the 0.01 significance level. TL: year-location + cultivar ID (baseline model), GW incorporates the main effects of SNPs and of EC. GW-G×W adds to GW interactions between SNPs and EC. TGW includes year-location, SNP, and EC effects. TGW-G×W expands TGW by adding SNP-EC interactions.

Figure 3 shows the average within-trial correlation between realized yield and CV predictions (letters indicate groups that are significantly different according to a paired *t*-test). The full model (TGW-G×W) achieved the highest within-trial correlation between predicted and realized yield (0.58, Fig. 3). The CV-correlations we obtained with our full model were slightly higher than the ones previously reported for wheat using a similar CV scheme^4,13,14^.

We also conducted a validation with cultivars assigned to folds; this mimics the problem of predicting the performances of a cultivars that were tested in any past trial^13^. In this new CV, the prediction performance was worse than when year-locations were assigned to folds. The baseline model yielded a slightly negative (−0.12, Supplementary Fig. 5) within year-location correlation. This happens because, within-trial, only genotype, genotype-by-location, and genotype-by-year-location can contribute to prediction accuracy. But these effects cannot be learned by the baseline model in a CV1 scheme. On the other hand, the models that included SNPs and ECs achieved a positive (albeit moderate) prediction correlation, and the relative ranking of the models was the same as the one observed in the leave-trial-out CV. Models GW-G×W and TGW-G×W achieved an average correlation of 0.25 (Supplementary Fig. 5). Previous studies^13^ have also reported low prediction accuracy when predicting phenotypes of untested cultivars. However, the reduction in prediction correlation was particularly marked in this data set because the cultivars included in it are not ‘close relatives’, and genomic prediction relies heavily on genetic relatedness^15^. Considering the low accuracy achieved in CV1 by the best performing model, we conclude that accurate prediction of within-year location performance requires, for the type of data that we considered, at least 1 year of testing per cultivar included in the simulation.

### Computer-simulated performances

The results presented thus far used only past trial data. In this section, we describe a computer-simulation platform that integrates those data with historical weather records.

**Fig. 4:**
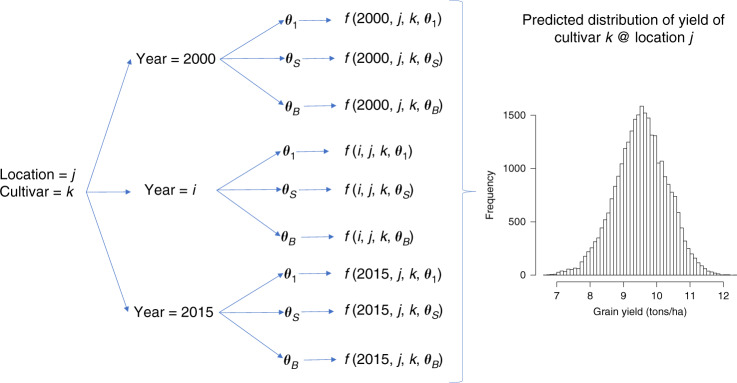
Monte Carlo simulation. Indices *i, j, k*, and *s* index year (2000–2015), location (*j* = 1,…, 16 locations), cultivar (i.e., the SNP genotypes, *k* *=* 1,…,28 cultivars) and sample from the posterior distribution of model parameters (*s* *=* 1,…,B, B = 20,000).

A conceptual description of the simulation process is presented in Fig. 4. We used the full model (TGW-G×W) to predict yield for 16 locations representative of French wheat-producing regions (Supplementary Fig. 4) and 28 wheat lines (***x***_*i*_; *i* *=* 1,…,28) for which we have extensive records from Arvalis’ network of trials. For each of the selected locations, we collected 16 years (2000–2015) of historical weather data. We used a crop model to derive environmental covariates (***w***_*ijk*_) for each year-location for which we had weather data. Using the BGLR software package^16^, we fitted model TGW-G×W to all the data from past field trials. The software was used to collect 100,000 samples from the posterior distribution of the parameters of model TGW-G×W. These samples were thinned at an interval of 5, producing 20,000 samples (***θ***_*s*_; *s* *=* 1,…, 20K). We then evaluated the prediction function (*f*(·) in Fig. 4; see “Methods” for details) for every genotype, location, year, and sample of the model parameters. The combination of 16 sites, 16 years, 28 genotypes, and 20,000 samples led to a total of 144.3 million simulated data points.

### Predictive analytics

**Fig. 5:**
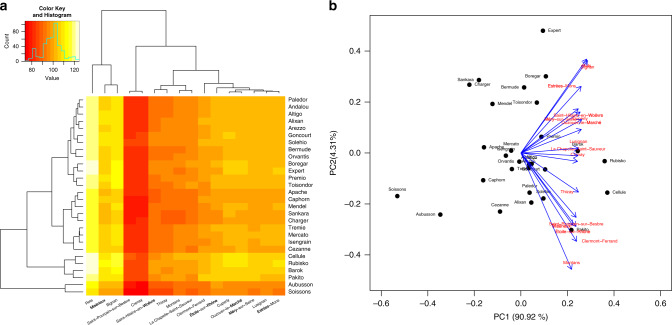
Predicted grain yield for selected genotypes in target locations. **a** Heatmap of expected grain yield (average from 16 years, 2000–2015 of weather data), and **b** biplot analysis of the predicted yield.

We used the simulated phenotypes to study the fitness of varieties to specific locations. Figure 5 shows a heatmap (left) and a biplot of the predicted average yields for the selected varieties at the target locations. Importantly, simulations are not restricted to the years in which each genotype was tested at each site. The heatmap in the margins of Fig. 5a shows dendrograms describing the clustering of locations and varieties. Celllule, Rubisko, Barok, and Pakito appeared as a cluster of high-yielding cultivars. On the other hand, historical varieties such as *Soissons* had relatively low-predicted yield across locations. The first two PCs of the matrix containing the predicted means by variety and location explained ~95% of the total variance of the cultivar means in the locations. Both the heatmap and the biplot can be used to identify varieties with high expected yield at each location.

**Fig. 6:**
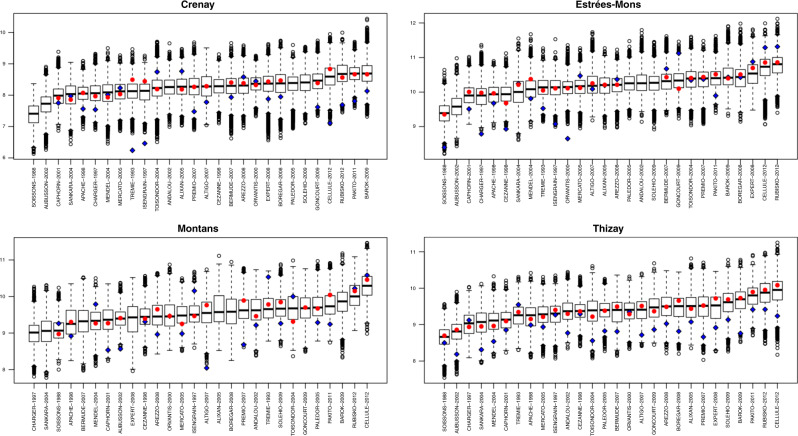
Distribution of simulated grain yield in selected cultivar–location combinations. The boxes summarize the predicted distribution of yields for each cultivar–location combination across 16 years of likely weather conditions (the line in the middle of the box represent the median, and the edges represent the 1st and 3rd quartiles). The red circles (blue diamond) represent the raw (BLUEs) genotype-by-location mean for the locations where each cultivar was tested.

The results in Fig. 5 are based on the average predicted yield at each of the 448 (16 × 28) cultivar–location combinations. The simulation also predicts the distribution of expected yield across possible weather conditions. This is displayed in Fig. 6 for four contrasting locations, including low (Crenay), intermediate (Thizay and Montans), and medium-high (Estrées-Mons) yield. In addition to differences in means (which can also be assessed in the heatmap of Fig. 5), the boxplots describe the variability in expected yields that can be attributed to differences in weather conditions, genotype-by-year-location, and uncertainty about model parameters. The results in Fig. 6 suggest that, compared to Crenay and Estrees-Mons, there is more uncertainty about average grain yield in Thizay and Montans. Likewise, the simulated performance can be used to quantify uncertainty about the yield performance of different varieties within each of the locations.

The red circles and blue diamonds in Fig. 6 represent the BLUEs (derived from the baseline model) and the raw means of grain yield of each cultivar–location combination, respectively. Since not all varieties were tested at all the locations, some location–cultivar combinations do not have BLUEs and raw means. There are important differences between the raw means, the BLUEs, and the median yield estimated from the simulation (represented by the central horizontal line in each of the boxes). The medians (and means) from the simulation are smoother than the raw means and the BLUEs because the simulation means are computed by averaging over 16 years of weather conditions and multiple configurations of model parameters. This is not the case of the raw means and the BLUEs, which are point-predictions derived using data from the years in which each cultivar was tested. The raw means, the BLUEs, and the simulated means produced distinct rankings of cultivars within each of the locations, which in turn can lead to different breeding decisions and variety recommendations.

Finally, we conducted a mean-stability analysis using a FW regression^6^ where the mean of the *k*th genotype on the *j*th location (*M*_*jk*_) was modeled as the sum of an environmental mean (*E*_j_), plus a cultivar-specific intercept (*b*_0*k*_), plus a regression on the environmental means, that is, $M_{jk} = b_{0k} + E_j + E_jb_{1k} + \delta _{jk}$; here, *b*_1*k*_ is a genotype-specific slope. We conducted these analyses using as data: (A) the raw genotype-by-trial means, (B) best linear unbiased estimates (BLUEs) of the same means derived from the baseline model, and (C) the variety-by-location means obtained by averaging the simulated results over 16 years of historical weather data.

**Fig. 7:**
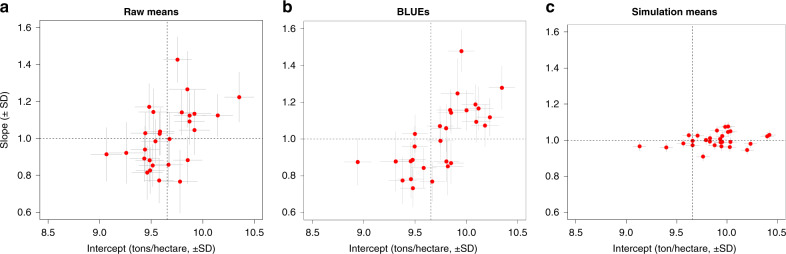
Finlay–Wilkinson coefficients. Coefficients were derived from the raw genotype-by-location means (**a**), BLUEs (**b**) and simulated genotype-by-location means averaged over 16 years of historical weather data (**c**). Vertical and horizontal lines, represent intervals defined as mean +/− posterior SD.

FW analyses based on the raw means (Fig. 7a) suggest substantial G × E, with estimated slopes ranging from 0.8 to 1.4. However, the same FW analyses based on BLUEs of the expected performance of a line in a location (Fig. 7b) showed much less variability in the slopes of the FW regressions. Results based on the simulated means (Fig. 7c) exhibited even smaller variability in slopes and much more precise estimates. Thus, the FW analyses based on the simulated means (which averaged predicted performances over 16 years of weather data for each of the locations) suggest small genotype-by-location variance at the level of the genotype’s mean in a location. This is consistent with the fact that, within this trial network, a sizable proportion of the environmental variance corresponds to year and year-location variance. The FW analysis in Fig. 7c identifies high-yielding varieties that are expected to perform well across many locations in French wheat-growing regions, including Cellule (intercept = 10.40, slope = 1.03) and Rubisco (intercept = 10.40, slope = 1.02).

To assess the stability of the FW results, we conducted 100 twofold cross-validations. Briefly, we split the observed and the simulated data of each cultivar into two folds. We then performed the FW regression analyses presented above within each of the folds. The averages (SEs) of the CV correlation between the predicted slopes of folds 1 and 2 were 0.342 (+/−0.015), 0.447 (+/−0.012), and 0.502 (+/−0.016), for the raw means, BLUEs, and the simulated means, respectively. In a paired *t*-test, the three correlations were significantly different (all the Holm’s adjusted *p*-values were smaller than 0.001).

## Discussion

Genetic-by-environment interactions (G×E) are a significant source of variance in crop phenotypes. The importance of G×E was recognized almost a century ago^17^. Since then, several statistical methods for the study of G×E were developed, including fixed-effects^18–21^ and mixed-effects^22,23^ models, reaction norms^24^, factor analytic methods^25^, and other reduced-rank methods^26^. More recently, G×E models that integrate DNA sequences, alone^13,14^ or in combination with ECs^4,27,28^ were developed and tested in multiple crops. However, a sizable fraction of the environmental and G×E variance is often due to year and year-location effects. This has limited the use of G×E models in breeding and research because predictions from such models require knowledge about future weather conditions which are mainly uncertain.

The concept of “target breeding environments” has been proposed as a way to incorporate G×E in breeding decisions^3^. However, predicting the performance of a cultivar in a target environment is challenging because, as noted, a sizable fraction of the environmental variance (50% in the data set analyzed in this study) corresponds to within-location year-to-year variation in weather conditions. Therefore, accurate prediction of the performance of genotypes at target environments would require testing genotypes over many years. This is both costly and inefficient because it delays breeding decisions, ultimately reducing genetic progress. On the other hand, when breeding decisions are based on the predicted performance derived from 1 or 2 years of testing, many genotypes are advanced without evaluating them under weather conditions that may greatly impact their performance (e.g., drought, heat, and cold stress).

We propose that computer simulations that integrate field trial data with DNA sequences and historical weather records can be used to tackle the longstanding problem of predicting genotypes’ future performance under largely uncertain weather conditions—a similar idea was used before by Chenu et al.^27^. However, the study by Chenu et al. simulated phenotypes assuming a crop model with known parameter values, simulated genotypes and selected environmental scenarios. Our approach builds upon Chenu’s work by proposing a simulation strategy that is heavily data-driven. We use real genotypes, historical weather data, and G×E patterns learned from field trial data without assuming an underlying crop model. Furthermore, rather than fixing parameter values (e.g., QTL effects) to point estimates and weather conditions to scenarios, our simulation strategy fully accounts for uncertainty about model parameters and uses historical weather data to account for likely weather conditions.

The continuous development of computing power and algorithms has led to increased interest in using computer simulations to assist breeding decisions and agronomic recommendations. However, the adoption of computer simulations in plant breeding remains limited because it is difficult to develop realistic simulations that can fully account for the complexity of how genomes respond to environmental conditions. There are often too many options as to how to simulate genotypes and phenotypes (e.g., how to model additive and non-additive effects, and how to simulate G×E) that make fully in-silico simulators mostly unrealistic. The approach used in this study overcomes these limitations by developing a computer-simulation platform that learns G×E patterns from past trial data and uses DNA sequences and historical weather records to simulate performances over possible environmental conditions. The simulation platform predicts not only the expected performance of a cultivar in a location but also the expected distribution of a trait over likely weather conditions. Importantly, the predictive distribution also accounts for uncertainty about model parameters.

Implementing the simulation approach proposed in this study requires (i) a model that integrates genetic and EC data, (ii) extensive multi-environment field testing data linked to genotypes and environmental covariates, and (iii) historical weather data.

For the model, we used a reaction norm for SNPs and ECs^4^; however, we highlight that the platform can be implemented with any (Bayesian) model that integrates DNA sequences and ECs, including crop models^5^.

The implementation of the proposed simulation platform requires extensive field trial data covering a diverse set of genotypes, many years, and locations. Access to such data is essential because those data are the source from where G×E patterns are learned. Ideally, the data should be dense enough to guarantee that simulations “interpolate” and do not “extrapolate”. Many public and private breeding organizations have generated large volumes of genetic and phenotypic data from field trials. The proposed simulation platform offers these organizations the opportunity to leverage their data with historical weather records to produce predictions of cultivars’ performances that are more robust than those that can be derived when varieties are tested for 1 or 2 years. However, we emphasize that the simulation platform presented here should not be conceived as a tool to predict performances outside of the genotype/environment space represented in the training data. Moreover, we do not recommend simulating phenotypes for untested cultivars, unless such varieties are closely related to the ones used to train the models.

Historical weather data, the third input in the simulation platform, should be readily accessible for locations with (or with nearby) weather stations. In this study, we applied the framework using historical weather data from individual locations. However, there are multiple potentially useful variants of this approach that are worth mentioning. First, if the goal is to breed or to produce agronomic recommendations for large growing regions, one could simulate at various locations and aggregate the simulated data at the regional level. Second, in this study, we used a long series of historical weather data. However, the same framework could be used as a tool for sensitivity analyses. For example, one could evaluate the effects of climate change by over-sampling years with adverse events (e.g., drought, heat stress) that may increase in frequency under a climate change model.

We used the proposed simulation platform to generate ~143 M grain yield values resulting from the combination of 28 genotypes at 16 locations, 16 years of historical weather data for each of those locations, and 20,000 possible configurations of model parameters. The simulated data were used to approximate the expected distribution of yield for 448 (28 × 16) genotype–location combinations. The mean-stability analysis conducted using the simulated phenotypes averages over likely weather conditions and suggests lower G×E levels in the cultivar–location means, than analyses based on raw-trial means or trial BLUEs. Therefore, the simulation platform can aid in identifying cultivars with consistently high yield across locations.

In conclusion, we presented a data-driven simulation platform that uses experimental data and historical weather records to predict cultivars’ future performances, accounting for uncertainty about future weather and model parameters. The resulting forecasts smooth-out variability attributable to year-location and genotype-by-year-location; therefore, they are more precise than the ones derived using a few years of testing.

## Methods

### Experimental data

Genotypes for each of 481 French-registered varieties were obtained using the Axiom high-density genotyping platform (Affymetrix Inc., Santa Clara, CA) containing 420K (K = 1,000) SNPs^7^ and generated within the Breedwheat project (ANR-10-BTR-03). Only SNPs with minor allele frequency >0.05, with a calling rate >0.8, and with <10% of heterozygous loci, were used in the analyses. A total of 167,440 SNPs fulfilled this criterion. The remaining missing genotypes were imputed to the mean (i.e., two times the allele frequency at the SNP).

Phenotype data originated in post-registration evaluation trials carried out each year by Arvalis for newly registered varieties. The trial network included 242 locations and a total of 752 year-locations. Each year-location had one (un-replicated) yield trial in which cultivars were arranged in a randomized complete block design with a plot size of 12 m². Sowing dates and densities were adjusted at each location to represent the usual practices at each location. Trials were managed to reduce any biotic or abiotic factor that may reduce grain yield potential (optimal nitrogen fertilization, weed, insect, and disease control). Plots were harvested at maturity using a combine harvester, and the grain yield of each plot was adjusted to 15% moisture.

A single check was used across all trials; however, since cultivars were tested at multiple year-locations, many genotypes provided strong connections between trials. To quantify this, we created a connectivity index (*T*_*ij*(*r*)_) that counted the number of trials connected to trial *ij* through at least *r* genotypes (*r* = 1,…,5). The average values of these indices in the data set were $\bar T_{\left( 2 \right)} = 573.3$, $\bar T_{\left( 3 \right)} = 473.3$, $\bar T_{\left( 4 \right)} = 402.3$, and $\bar T_{\left( 5 \right)} = 339.8$. Histograms of the distribution of these indices are presented in Supplementary Fig. 1.

Environmental covariates were chosen based on physiological knowledge of bread wheat response to environmental factors (water, radiation, temperature) at different periods of the crop cycle (see Supplementary Table 1 for a list of ECs).

Weather data were gathered from weather stations located within 10 km of the trial. The crop cycle was divided into phases between sowing, emergence, the beginning of stem elongation, meiosis, heading, anthesis, milky, and maturity corresponding to growth stages (GS) 00, 10, 30, 39, 55, 65, 75, 92^29^. The GS dates were simulated using an ecophysiological model based on the daily accumulation of thermal time, possibly modified by vernalization and photoperiod factors^30–32^.

Temperature accumulation was calculated daily using a piece-wise linear function with three knots or cardinal temperatures (0, 24, and 35 °C). Accumulation of temperature was nil below 0 °C or above 35 °C; it increased linearly between 0 and 24 °C, and decreased linearly between 24 and 35 °C. Emergence reached after 150 °C—days from sowing with no effect of photoperiod or vernalization.

From emergence to GS30, temperature accumulation was reduced by vernalization and photoperiod factors both varying between 0 and 1. The photoperiod factor was calculated as ${\mathrm{PF}} = \left( {P_H - P_{{\mathrm{base}}}} \right)/\left( {P_{{\mathrm{opt}}} - P_{{\mathrm{base}}}} \right)$ where *P*_*H*_ is the daily photoperiod (in hours) and *P*_base_ and *P*_opt_ are parameters equal to 6.3 and 20 h, respectively. The vernalization factor was calculated as ${\mathrm{VF}} = \left( {{\mathrm{VDD}} - V_{{\mathrm{base}}}} \right)/\left( {V_{{\mathrm{sat}}} - V_{{\mathrm{base}}}} \right)$, where VDD is the number of accumulated “vernalizing days” and *V*_base_ and *V*_sat_ are parameters equal to 0 and 45, respectively. VDD was calculated daily using a piece-wise linear function of daily mean temperature defined by three cardinal temperatures of −1, 6, and 17 °C. Accumulation of vernalizing days was nil when daily mean temperature was ≤−1 °C or ≥17 °C; it increased linearly between −1 and 6 °C, was equal to 1 at 6 °C, and decreased linearly between 6 and 17 °C. From GS30 to GS55, the base temperature was set at 3.5 °C, *P*_base_ was set at 7.7 h, and the accumulation of thermal time was limited by the photoperiod factor only. Two cultivar-dependent parameters (GDD_pv_ = growing degree days reduced by photoperiod and vernalization factors, and GDD_p_ = growing degree days reduced by the photoperiod factor) determined the accumulation of modified thermal time required from emergence to GS30 and from GS30 to GS55, respectively. GS39 was determined using backward calculation from GS55 as GS39 = GS55–1.2 × *Phyll*, with GS55 representing the heading date in degree days since sowing, and *Phyll* representing the phyllotherm parameter, which was calculated as follows:

$$Phyll = \left[ {100/\frac{{2.54 \times (P_{{\mathrm{GS}}10} - P_{{\mathrm{GS}}00})}}{{\left( {{\mathrm{GDD}}_{{\mathrm{GS}}00 - {\mathrm{GS}}10} + \frac{{14.396}}{{Rg_{{\mathrm{GS}}00 - {\mathrm{GS}}10}}}} \right) + 1.0104}}} \right] \times 0.9393 + 0.000379 \times d_{{\mathrm{GS}}10}$$

Above, *P*_GS10_ and *P*_GS00_ are the photoperiods at GS10 and GS00, respectively; GDD_GS00–GS10_ represent growing degree days between GS00 and GS10; *Rg*_GS00–GS10_ is the average global radiation (joules.cm^−2^) between GS00 and GS10. Finally, *d*_GS10_ represents the plant density at GS10. The phyllotherm parameter was bound to the 66–120 range.

Following GS55, only temperature affected plant development. GS65 was calculated as as GS65 = GS55 + 0.05899 × (1.596 × GS55–93.61), where GS65 and GS55 correspond to anthesis and heading dates in degree days since sowing, respectively. Then, GS75 reached after 430 days from GS55, and GS92 reached after 770 days from GS55.

### Statistical models

The baseline model (TL) included an intercept, plus the normal, independent and identically distributed (NIID) random effects of the year, $Y_i\sim {\mathrm{NIID}}(0,\sigma _Y^2)$, location, $L_j\sim {\mathrm{NIID}}(0,\sigma _L^2)$, year-location, $YL_{ij}\sim {\mathrm{NIID}}(0,\sigma _{YL}^2)$, and cultivar, $V_k\sim {\mathrm{NIID}}(0,\sigma _V^2)$; therefore,

4 $${\mathrm{TL{:}}}\,y_{ijk} = \mu + Y_i + L_j + YL_{ij} + V_k + \varepsilon _{ijk},$$

where $$\varepsilon _{ijk}\sim {\mathrm{NIID}}\left( {0,\sigma _\varepsilon ^2} \right).$$

The GW model was obtained by substituting the cultivar, year, location, and year-location effects with genetic and environmental random effects linked to SNPs and ECs, respectively. Specifically, we replaced the cultivar effect with a multivariate normal random effect,${\boldsymbol{V}} = \left( {V_1,\ldots,V_{482}} \right)\prime$, which had a covariance matrix proportional to ${\boldsymbol{G}} = {\boldsymbol{XX}}\prime /p$, that is, ${\boldsymbol{V}}\sim {\mathrm{MVN}}({\mathbf{0}},{\boldsymbol{G}}\sigma _V^2)$. Here ***X*** is a matrix of (centered and scaled) SNP genotypes (*p* is the number of SNPs). Likewise, we replaced the year, location, and year-location effects in Eq. (1) with a multivariate normal random effect that had a covariance structure derived from the ECs: ${\boldsymbol{w}} = \left\{ {w_{ijk}} \right\}\sim {\mathrm{MVN}}({\mathbf{0}},{\mathbf{\Omega}}\sigma _{EC}^2)$, where ${\mathbf{{\Omega} }} = {\boldsymbol{WW}}\prime /q$. Here, ***W***_*n*×*q*_ is a matrix of (centered and scaled) environmental covariates, and *q* is the number of environmental covariates. Therefore, the equation for the GW model was

5 $${\mathrm{GW{:}}}y_{ijk} = \mu + V_k + w_{ijk} + \varepsilon _{ijk}.$$

Subsequently, we expanded the GW model by adding interactions between SNPs and ECs. Specifically, we introduced a random effect ${\boldsymbol{VW}} = \{ VW_{ijk}\} \sim {\mathrm{MVN}}\left( {{\mathbf{0}},({\boldsymbol{Z}}_g{\boldsymbol{GZ}}_g^\prime )\# {\boldsymbol{\Omega}} \sigma _{V \times EC}^2} \right)$^4^. Here, ***Z***_*g*_ is a design matrix connecting phenotypes with cultivars, and ***G*** and **Ω** are genomic and environmental relationship matrices, respectively. Above, “#” represents the Hadamard (or cell-by-cell) product between two matrices, and $\sigma _{V \times EC}^2$ is a variance parameter associated with the interaction term. Thus,

6 $${\mathrm{GW}} - {\mathrm{G}}\,\times{\mathrm{W}{:}}y_{ijk} = \mu + V_k + w_{ijk} + VW_{ijk} + \varepsilon _{ijk}.$$

Variance component estimates revealed that the GW and GW-G×W models did not fully capture the environmental variance captured by the TL model. Therefore, to fully capture between-trial differences, we added back year and location effects, without (TWG) and with (TGW-G×W) interactions between SNPs and ECs, that is:

7 $${\mathrm{TWG{:}}}\;y_{ijk} = \mu + Y_i + L_j + V_k + YL_{ij} + w_{ijk} + \varepsilon _{ijk},$$

and

8 $${\mathrm{TGW}} - {\mathrm{G}}\,\times{\mathrm{W}{:}}\;y_{ijk} = \mu + Y_i + L_j + YL_{ij} + V_k + w_{ijk} + VW_{ijk} + \varepsilon _{ijk}.$$

The distributional assumptions for each of the terms in Eqs. (4) and (5) were as described before (see models TL, GW, and GW-G×W).

### Assessment of prediction accuracy

We used a 10-fold CV with trials (i.e., year-location IDs) assigned to folds to assess prediction accuracy. We chose the within-year-location correlation between predictions and observed yield as a metric to assess prediction accuracy. Thus, from a 10-fold CV we had as many correlations (*r*) per model as year-locations were represented in the data (767). To assess statistical differences between models, we employed a paired *t*-test applied to Fisher’s z-transform, that is, $z = \frac{{\sqrt {n_{ij} - 3} }}{2}\log ((1 + r)/(1 - r)),$ with *n*_*ij*_ being the number of records in the particular year-location. *P*-values were derived using the *t*-test function of R. These *p*-values were used to group the models according to their predictive power using the orderPValue function in the agricolae R-package^33^ with *α* = 0.05.

### Simulation

A conceptual description of the simulation algorithm is presented in Fig. 4. Using the TGW-G×W Eq. (4), we simulated performances for 28 genotypes that are well-represented in Arvalis’ trial network and 16 locations representative of French wheat-producing regions (Supplementary Fig. 4). For each location, we retrieved 16 years of historical weather data (from 2000 to 2015) and used those data to derive ECs for each for the 6720 (16 × 16 × 28) year–location–cultivar combinations represented in the simulation. Subsequently, we used 20,000 samples from the posterior distribution of the parameters of the TGW-G×W model to evaluate, for each of the sample–genotype–year–location combinations, the prediction function:

9 $$f\left( {i,j,k,\theta _s} \right) = \mu _{(s)} + L_{j(s)} + V_{k(s)} + w_{ijk(s)} + VW_{ijk(s)}$$

Above, *i* indicates years (2000–2015), *j* location, *k* cultivar, and *s* sample from the posterior distribution of the model parameters (***θ***_*s*_). Note that the prediction Eq. (6) uses (from the regression model TGW-G×W) only the terms that can be learned from past data (*μ*_(*s*)_ + *L*_*j*(*s*)_) or predicted from knowledge of model parameters, SNPs, and ECs (*V*_*k*(*s*)_ + *w*_*ijk*(*s*)_ + *VW*_*ijk*(*s*)_). Thus, predictions from the simulation did not include year and year-location effects that cannot be predicted from knowledge of ECs. Equation (6) was evaluated for each genotype–location–year–sample combination, thus producing ~143.4 million simulated data points.

### Biplots

We analyzed the simulated data using the site regression (SREG) model^2,23^. As the response variable, we used the mean of simulated grain yield for variety *i*, location *j*^34,35^.

### Finlay–Wilkinson (FW) regressions

We conducted means-stability analyses using an FW regression^6^ of the form $M_{jk} = b_{0k} + E_j + E_jb_{1k} + \delta _{jk}$, where *M*_*jk*_ are the means of genotype *k* in location *j*, $E_j\sim {\mathrm{NIID}}\left( {0,\sigma _E^2} \right)$, ${\boldsymbol{b}}_0 = \left\{ {b_{0k}} \right\}\sim {\mathrm{MVN}}\left( {{\mathbf{0}},{\boldsymbol{G}}\sigma _{b_0}^2} \right)$, and ${\boldsymbol{b}}_1 = \left\{ {b_{1k}} \right\}\sim {\mathrm{MVN}}\left( {{\mathbf{0}},{\boldsymbol{G}}\sigma _{b_1}^2} \right)$ are the location means and cultivar-specific intercepts and slopes, respectively. We implemented the FW regressions in two steps: (i) in the first one we estimated environmental means using a random-effects additive model of the form $M_{jk} = \tilde V_k + \tilde E_j + \tilde \delta _{jk}$, and (ii) subsequently, we inferred intercepts and slopes using the FW model with *E*_*j*_ replaced by $\tilde E_j$. Both steps were implemented using the BGLR R-package (see Supplementary Software).

The results in Fig. 7 are based on an analysis of the entire data set. The dots are the estimated means for the intercepts and slopes, and the vertical and horizontal lines are the estimated posterior standard deviations. We also conducted 100 two-fold cross-validations in which we divided the observed (in the case of the raw means and BLUP method) or the simulated data of each cultivar into two halves. Subsequently we applied FW regressions to each of the halves and with this we estimated the correlation between the slopes inferred in each of the halves. This was repeated 100 times, and statistical differences were assessed using paired *t*-tests.

### Software

Data analyses were performed using the R Statistical package^36^. Models were fitted using the BGLR package^16^. All the models were fitted with the default hyper-parameter values chosen by BGLR. The SREG model and the biplots were generated using custom R-scripts. The scripts used to fit the models and perform the biplot analyses and FW regressions are provided in the Supplementary Software.

### Reporting summary

Further information on research design is available in the Nature Research Reporting Summary linked to this article.

## Supplementary information

Supplementary Information

Supplementary Software

Description of Additional Supplementary Files

Reporting Summary

## Data Availability

The data are not publicly available due to them containing proprietary information. However, the data that support the findings of this study are available upon request. Specific data transfer agreements may be required for each individual request.

## Code availability

The scripts used to fit the models and perform the biplot analyses are provided, together with a sample data set, in the Supplementary Software.
