# Author Correction: A data-driven simulation platform to predict cultivars’ performances under uncertain weather conditions

**DOI:** 10.1038/s41467-021-22384-w

**Published:** 2021-03-24

**Authors:** Gustavo de los Campos, Paulino Pérez-Rodríguez, Matthieu Bogard, David Gouache, José Crossa

**Affiliations:** 1 Departments of Epidemiology & Biostatistics and Statistics & Probability, IQ - Institute for Quantitative Health Science and Engineering, Michigan State University, East Lansing, MI USA; 2 Colegio de Postgraduados, CP 56230 Montecillos, Estado de México, México; 3 Arvalis – Institut du Végétal, 6 Chemin de la côte vieille, 31450 Baziège, France; 4 Arvalis – Institut du Végétal, Station Expérimentale, 91720 Boigneville, France; 5 International Maize and Wheat Improvement Center (CIMMYT), Km 45, Carretera México-Veracruz, El Batán, Texcoco, Edo. de México, México; 6 Present Address: Terres Inovia, 11 rue Gaspard Monge, 33600 Pessac, France

**Keywords:** Data integration, Agricultural genetics

Correction to: *Nature Communications* 10.1038/s41467-020-18480-y, published online 25 September 2020.

The original version of this Article omitted from the Acknowledgments the following text: ‘The meteorological data were obtained from Météo France, Réseau agroclimatique INRAe and Arvalis Institut du Végétal’. This has now been corrected in both the PDF and HTML versions of the Article.

