# Supplementary material for: A data-driven simulation platform to predict cultivars’ performances under uncertain weather conditions
Source: Nat Commun. 2020 Sep 25;11:4876. doi: 10.1038/s41467-020-18480-y (PMC7519145; doi:10.1038/s41467-020-18480-y)
Supplement: Supplementary file 1 — Supplementary Information [file 41467_2020_18480_MOESM1_ESM.pdf]

Supplementary Information

A data-driven simulation platform to predict cultivars' performances under uncertain weather conditions

Gustavo de los Campos[1,*], Paulino Pérez-Rodríguez[2*], Matthieu Bogard[3*], David Gouache[4], & José Crossa[2,5]

Supplementary Figures

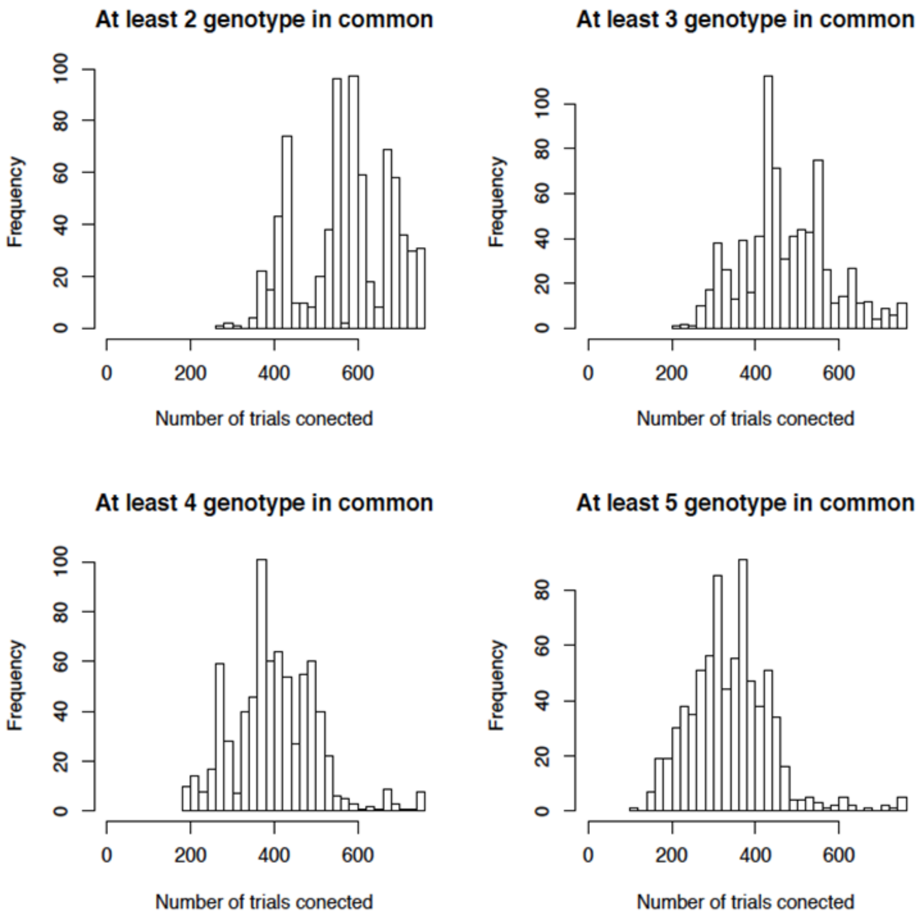

Supplementary Figure 1. Distribution of the number of trials connected through at least *r* genotypes (*r*=2,3,4,5)

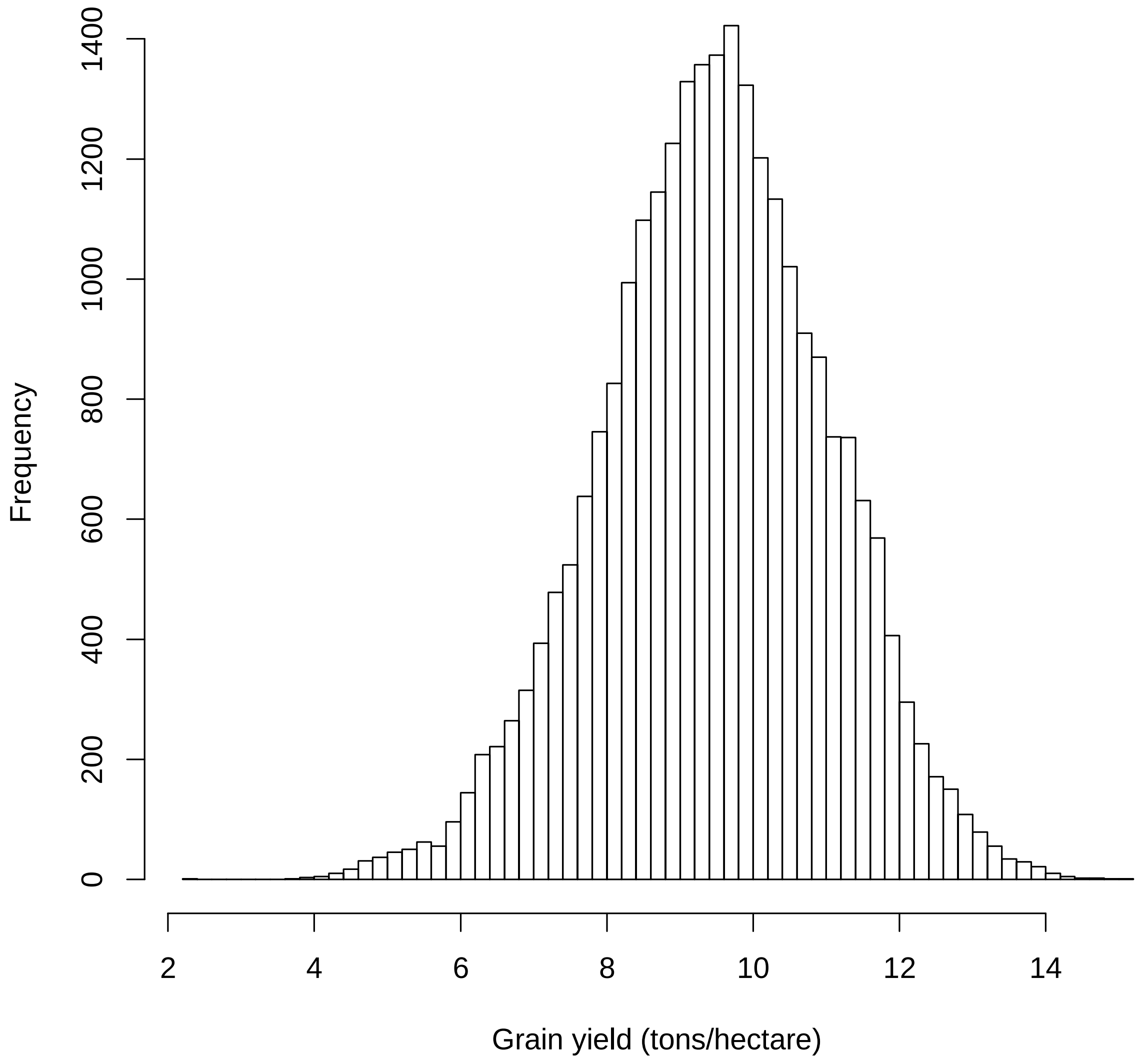

Supplementary Figure 2. Histogram of grain yield.

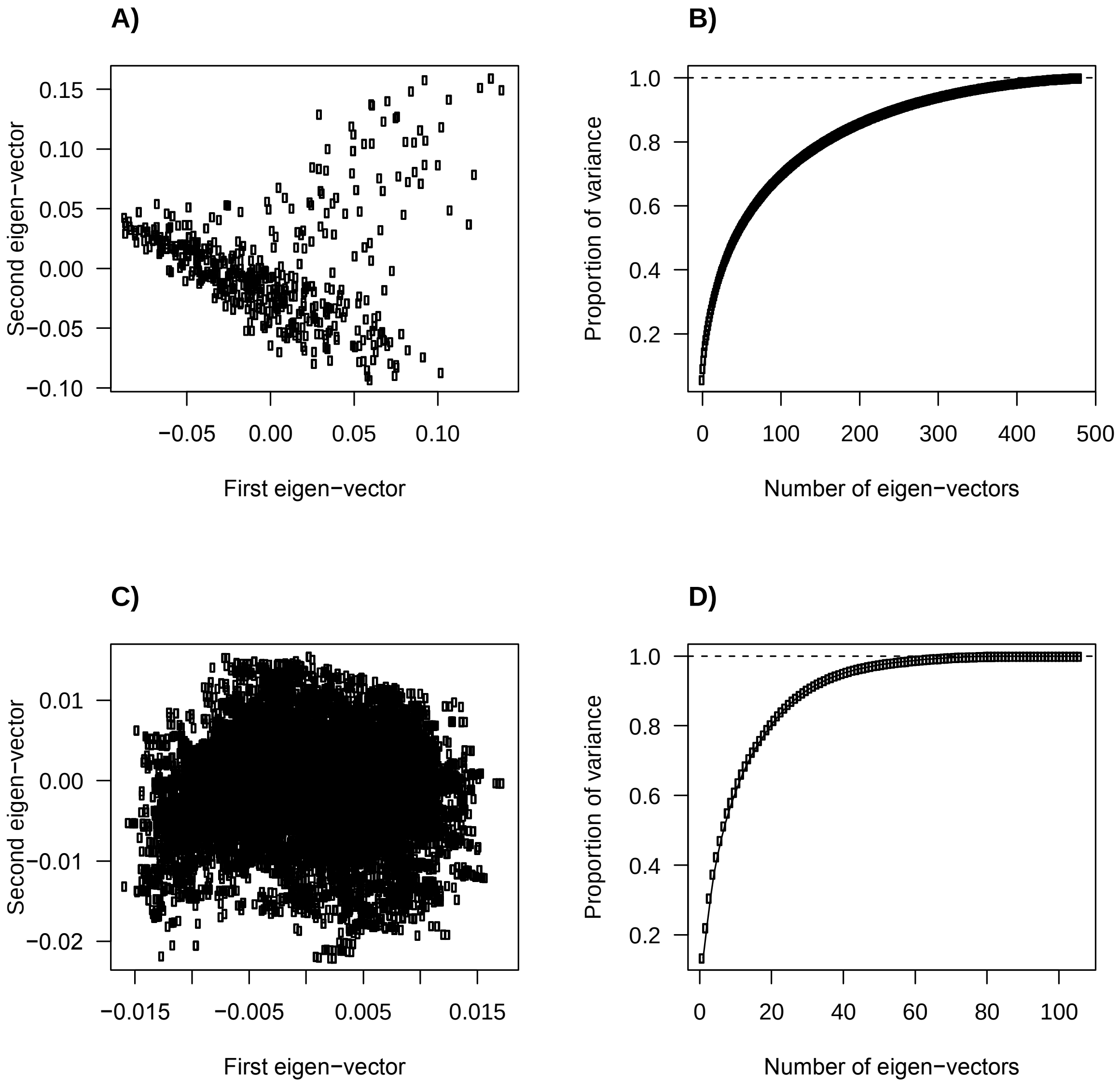

Supplementary Figure 3. First two eigen-vectors (left) and eigenvalues (right) of the genomic (top row, A and B) and environmental (bottom row, C and D) relationship matrices.

A)

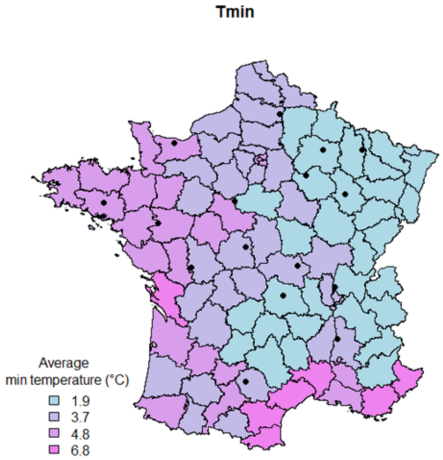

B)

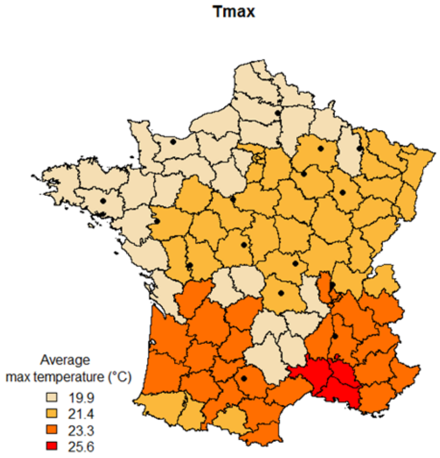

C)

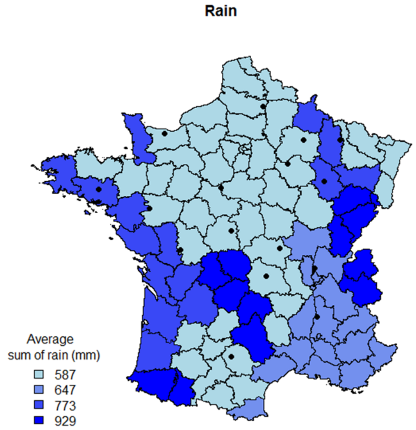

D)

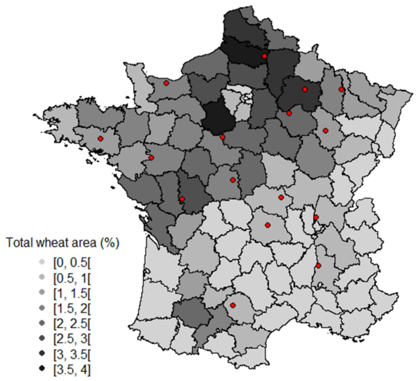

Supplementary Figure 4: Climate analysis and clustering of France regions for minimum (A), maximum temperatures (B) and rainfall (C) and percentage of total wheat area (D). One representative location was chosen for each region and average daily temperatures or rainfall were calculated over 20 years of historical weather data (1986-2017). Ward clustering into four groups was performed on Euclidean distances calculated between locations for average daily minimum temperatures from the 1st of October to the 1st of April, for average daily maximum temperatures from the 1st of April to the 31st of July

and for average daily rainfalls over the whole growing period (from the 1st of October to the 31st of July). Locations selected for the simulation study are shown in black or red dots.

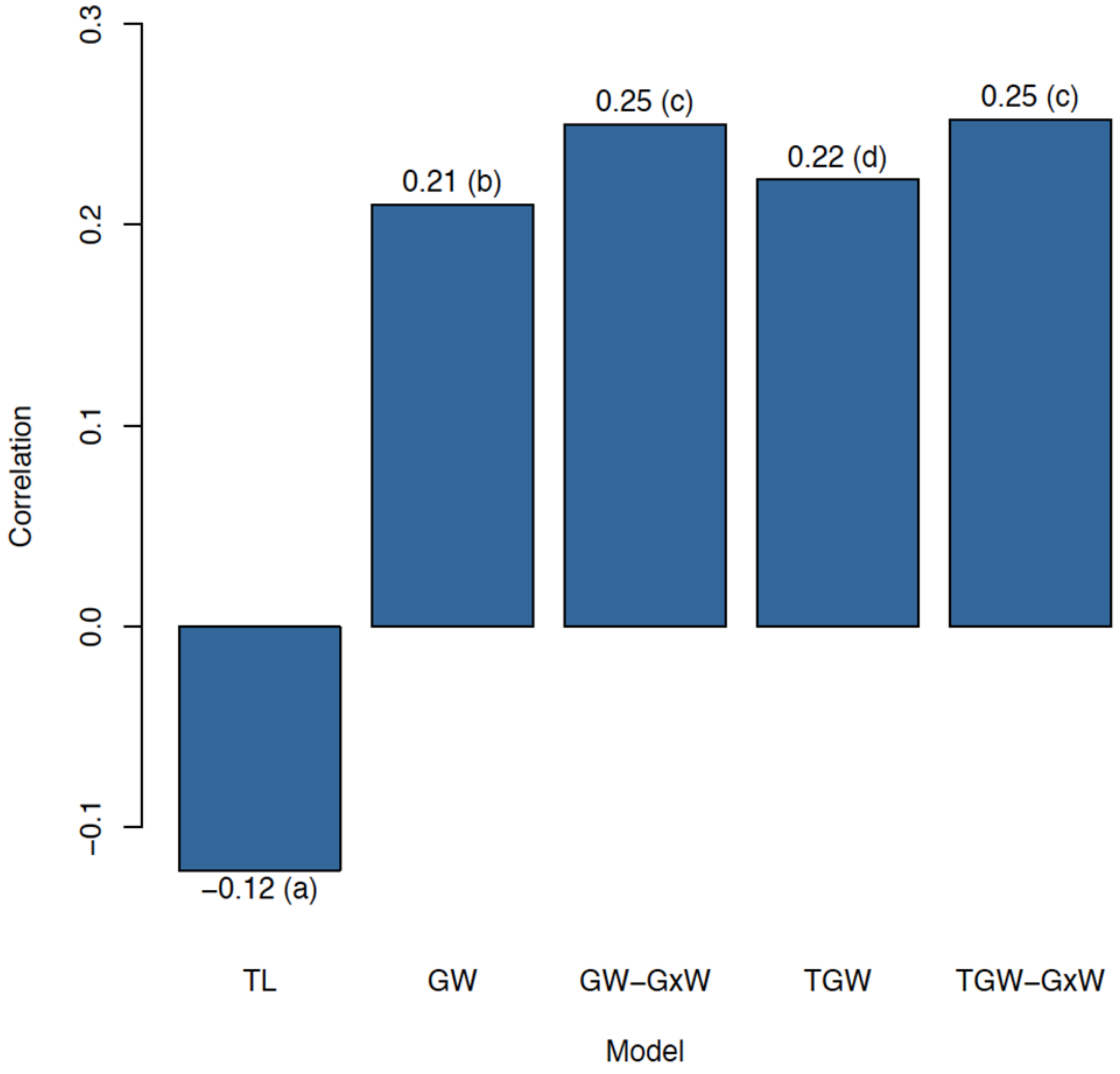

Supplementary Figure 5. Average within-trial correlation between predicted and observed yield obtained in a cross-validations in which cultivars were assigned to folds. (Different letters indicate differences at the 0.01 significance level). TL: year-location + cultivar ID (baseline model), GW incorporates the main effects of SNPs and of EC. GW-GxW

adds to GW interactions between SNPs and EC. TGW includes year-location, SNP, and EC effects. TGW-GxW expand TGW by adding SNP-EC interactions. (See Figure 3 for results obtained when year-locations were assigned to folds.)

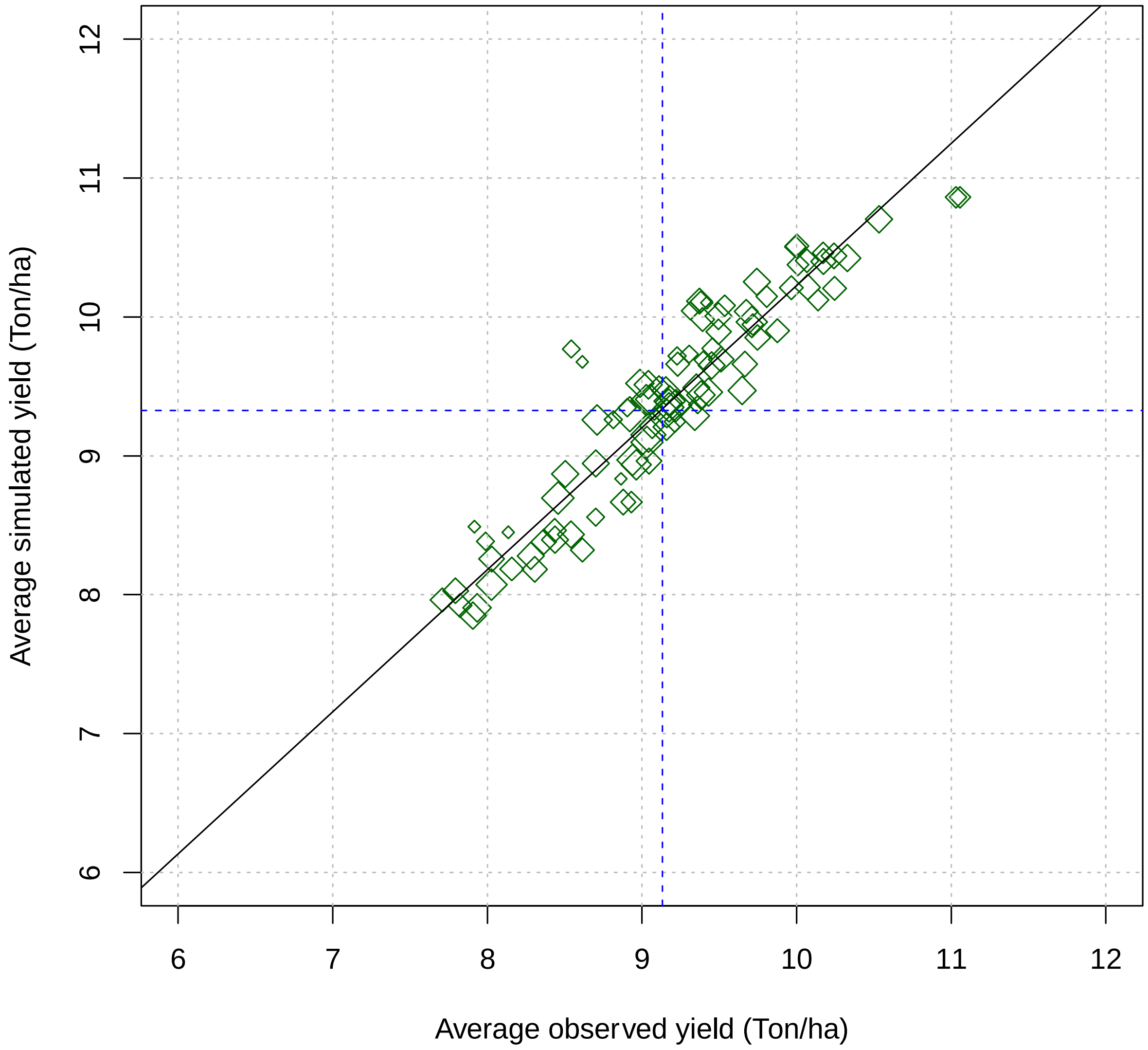

Supplementary Figure 6. Average observed yield vs average simulated yield. Each square represents a genotype in a location, the size of the square is proportional to the logarithm of the number of records, the vertical and horizontal dashed lines are the average of the variables in the vertical and horizontal axis.

Supplementary Table 1: Environmental Covariates List and Description

Code	Category	Definition
sTmin.12ST	frost	sum of daily minimum temperatures below -12°C from sowing to the start of tillering
nbj.12ST	frost	number of days with minimum temperatures below -12°C from sowing to the start of tillering
nbj0Ep1M	frost	number of days with minimum temperatures below 0°C from the beginning of stem elongation to meiosis
sTmin4Ep1M	frost	sum of daily minimum temperatures below -4°C from meiosis to the beginning of stem elongation
sTmin4MM	frost	sum of daily minimum temperatures below 4°C between meiosis - 10 days to meiosis + 10 days
sTmin4EF	frost	sum of daily minimum temperatures below 4°C from heading to flowering
sTmin4FL	frost	sum of daily minimum temperatures below 4°C from meiosis to milky stage
nbj4Ep1M	frost	number of days with minimum temperatures below 4°C from meiosis to the beginning of stem elongation
nbj4MM	frost	number of days with minimum temperatures below 4°C between meiosis - 10 days to meiosis + 10 days
nbj4EF	frost	number of days with minimum temperatures below 4°C from heading to flowering
nbj4FL	frost	number of days with minimum temperatures below 4°C from flowering to milky stage
nbj.5SEp1	frost	number of days with minimum temperatures below 5°C from sowing to the beginning of stem elongation
nbj5ME	frost	number of days with minimum temperatures below 5°C from meiosis to heading
sTmax25Ep1M	heat	sum of daily maximum temperatures above 25°C from meiosis to the beginning of stem elongation
sTmax25ME	heat	sum of daily maximum temperatures above 25°C from meiosis to heading
sTmax25MM	heat	sum of daily maximum temperatures above 25°C between meiosis - 10 days to meiosis + 10 days
sTmax25FL	heat	sum of daily maximum temperatures above 25°C from flowering to milky stage
sTmax25EL	heat	sum of daily maximum temperatures above 25°C from heading to milky stage
sTmax25LMat	heat	sum of daily maximum temperatures above 25°C from milky stage to maturity
sTmax30FF	heat	sum of daily maximum temperatures above 30°C between flowering - 6 days and flowering + 6 days
nbj25Ep1M	heat	number of days with maximum temperatures above 25°C from meiosis to the beginning of stem elongation

(continues next page)

(continued from previous page)

Code	Category	Definition
nbj25ME	heat	number of days with maximum temperatures above 25°C from meiosis to heading
nbj25MM	heat	number of days with maximum temperatures above 25°C between meiosis – 10 days and meiosis + 10 days
nbj25FL	heat	number of days with maximum temperatures above 25°C from flowering to milky stage
nbj25EL	heat	number of days with maximum temperatures above 25°C from heading to milky stage
nbj25Lmat	heat	number of days with maximum temperatures above 25°C from milky stage to maturity
Nb.j.Ep1.15	phenology	number of days between stem at 10mm above the tillering plateau and stem at 15mm above the tillering plateau
Nb.j.Ep1.15E	phenology	number of days between stem at 15mm above the tillering plateau and heading
QSEp1	photothermal ratio	ratio of the sum of global radiation over the sum of mean daily temperature from sowing to the beginning of stem elongation
Qep1M	photothermal ratio	ratio of the sum of global radiation over the sum of mean daily temperature from the beginning of stem elongation to meiosis
QME	photothermal ratio	ratio of the sum of global radiation over the sum of mean daily temperature from meiosis to heading
QFL	photothermal ratio	ratio of the sum of global radiation over the sum of mean daily temperature from flowering to milky stage
QEL	photothermal ratio	ratio of the sum of global radiation over the sum of mean daily temperature from heading to milky stage
QLMat	photothermal ratio	ratio of the sum of global radiation over the sum of mean daily temperature from milky stage to maturity
QMM	photothermal ratio	ratio of the sum of global radiation over the sum of mean daily temperature between meiosis – 10 days to meiosis + 10 days
Qannee	photothermal ratio	gratio of the sum of global radiation over the sum of mean daily temperature from sowing to maturity
RgSEp1	radiation	sum of global radiation from sowing to the beginning of stem elongation
RgEp1M	radiation	sum of global radiation from the beginning of stem elongation to meiosis
RgME	radiation	sum of global radiation from meiosis to heading
RgFL	radiation	sum of global radiation from flowering to milky stage
RgEL	radiation	sum of global radiation from heading to milky stage
RgLMat	radiation	sum of global radiation from milky stage to maturity
RgMM	radiation	sum of global radiation between meiosis – 10 days to meiosis + 10 days
sRg…180MM	radiation	sum of global radiation < 180 W.m-2 between meiosis – 10 days and meiosis + 10 days
nb.j.RG.180MM	radiation	number of days with global radiation < 180 W.m-2 between meiosis – 10 days and meiosis + 10 days

(continues next page)

(continued from previous page)

Code	Category	Definition
sRg.200MM	radiation	sum of global radiation < 200 W.m-2 between meiosis - 10 days and meiosis + 10 days
nb.j.Rg.200MM	radiation	number of days with global radiation < 200 W.m-2 between meiosis - 10 days and meiosis + 10 days
nb.j.Rg.200FF	radiation	number of days with global radiation < 200 W.m-2 between flowering - 6 days and flowering + 5 days
sTb0SEp1	temperature	sum of daily mean temperature above zero from sowing to the beginning of stem elongation
sTb0Ep1M	temperature	sum of daily mean temperature above zero from the beginning of stem elongation to meiosis
sTb0ME	temperature	sum of daily mean temperature above zero from meiosis to heading
sTb0FL	temperature	sum of daily mean temperature above zero from flowering to milky stage
sTb0EL	temperature	sum of daily mean temperature above zero from heading to milky stage
sTb0LMat	temperature	sum of daily mean temperature above zero from milky stage to maturity
sTb0MM	temperature	sum of daily mean temperature above zero between meiosis - 10 days to meiosis + 10 days
Tmoyb0SEp1	temperature	average daily mean temperature above zero from sowing to the beginning of stem elongation
Tmoyb0Ep1M	temperature	average daily mean temperature above zero from the beginning of stem elongation to meiosis
Tmoyb0ME	temperature	average daily mean temperature above zero from meiosis to heading
Tmoyb0FL	temperature	average daily mean temperature above zero from flowering to milky stage
Tmoyb0EL	temperature	average daily mean temperature above zero from heading to milky stage
Tmoyb0LMat	temperature	average daily mean temperature above zero from milky stage to maturity
Tmoyb0MM	temperature	average daily mean temperature above zero between meiosis - 10 days to meiosis + 10 days
Tmoyb0Annee	temperature	average daily mean temperature above zero from sowing to maturity
SpSEp1	water	sum of rainfall from sowing to the beginning of stem elongation
SpEp1M	water	sum of rainfall from the beginning of stem elongation to meiosis
SpME	water	sum of rainfall from meiosis to heading
SpFL	water	sum of rainfall from flowering to milky stage
SpEL	water	sum of rainfall from heading to milky stage

(continues next page)

(continued from previous page)

Code	Category	Definition
SpLMat	water	sum of rainfall from milky stage to maturity
SpMM	water	sum of rainfall between meiosis - 10 days to meiosis + 10 days
drainST	water	sum of water drainage from sowing to the start of tillering
drainTEp1	water	sum of water drainage from the start of tillering to the beginning of stem elongation
drainSEp1	water	sum of water drainage from sowing to the beginning of stem elongation
drainEp1M	water	sum of water drainage from the beginning of stem elongation to meiosis
drainME	water	sum of water drainage from meiosis to heading
jdrainST	water	number of days with drainage from sowing to the start of tillering
jdrainTEp1	water	number of days with drainage from the start of tillering to the beginning of stem elongation
jdrainEp1M	water	number of days with drainage from the beginning of stem elongation to meiosis
jdrainME	water	number of days with drainage from meiosis to heading
SomETM_ST	water	sum of daily maximum evapotranspiration from sowing to the start of tillering
SomETM_TEp1	water	sum of daily maximum evapotranspiration from the start of tillering to the beginning of stem elongation
SomETM_SEp1	water	sum of daily maximum evapotranspiration from sowing to the beginning of stem elongation
SomETM_Ep1M	water	sum of daily maximum evapotranspiration from the beginning of stem elongation to meiosis
SomETM_ME	water	sum of daily maximum evapotranspiration from meiosis to heading
SomETM_EL	water	sum of daily maximum evapotranspiration from heading to milky stage
SomETM_Lmat	water	sum of daily maximum evapotranspiration from milky stage to maturity
SomETR_ST	water	sum of daily real evapotranspiration from sowing to the start of tillering
SomETR_TEp1	water	sum of daily real evapotranspiration from the start of tillering to the beginning of stem elongation
SomETR_SEp1	water	sum of daily real evapotranspiration from sowing to the beginning of stem elongation
SomETR_Ep1M	water	sum of daily real evapotranspiration from the beginning of stem elongation to meiosis
SomETR_ME	water	sum of daily real evapotranspiration from meiosis to heading
SomETR_EL	water	sum of daily real evapotranspiration from heading to milky stage
SomETR_Lmat	water	sum of daily real evapotranspiration from milky stage to maturity
dhST	water	sum of water deficit from sowing to the start of tillering
dhSEp1	water	sum of water deficit from sowing to the beginning of stem elongation
dhEp1M	water	sum of water deficit from the beginning of stem elongation to meiosis

(continues next page)

Code	Category	Definition
dhME	water	sum of water deficit from meiosis to heading
dhFL	water	sum of water deficit from flowering to milky stage
dhLMat	water	sum of water deficit from milky stage to maturity
EausolEpi1	water	soil water status at the beginning of stem elongation
indST	water	ratio of real evapotranspiration over maximal evapotranspiration from sowing to the beginning of tillering
indSEp1	water	ratio of real evapotranspiration over maximal evapotranspiration from the beginning of tillering to the beginning of stem elongation
indEp1M	water	ratio of real evapotranspiration over maximal evapotranspiration fromthe beginning of stem elongation to meiosis
indME	water	ratio of real evapotranspiration over maximal evapotranspiration from meiosis to heading date
indEL	water	ratio of real evapotranspiration over maximal evapotranspiration from heading date to milky stage
indLmat	water	ratio of real evapotranspiration over maximal evapotranspiration from milky stage to maturity
