# Supplementary material for: A data-driven simulation platform to predict cultivars’ performances under uncertain weather conditions
Source: Nat Commun. 2020 Sep 25;11:4876. doi: 10.1038/s41467-020-18480-y (PMC7519145; doi:10.1038/s41467-020-18480-y)
Supplement: Supplementary file 2 — Supplementary Software [file 41467_2020_18480_MOESM2_ESM.zip › CODE/README.html]

README


# README

#### G. de los Campos and P. Perez-Rodriguez

#### 03/23/2020

## System requirements

All analyses were perfomed in a workstation with an Intel(R) Xeon(R) CPU E5-2680 v4 @ 2.40GHz with 64 Gb of RAM memory. The operating system installed in the workstation was Linux Centos 7, with kernel 3.10.0-957.5.1.el7.x86\_64. The statistical analysis were perfomed using R-3.6.3 and the following R packages and versions:

- BGLR version 1.0.8.
- agricolae version 1.3-3.
- doBy, version 4.6.6.
- gplots, version 3.0.3.

Altought the analyses were perfomed in the workstation described above, the four R-packages are available for linux, windows, and Mac OS.

## Installation guide

The R software can be downloaded from R webpage, http://www.r-project.org, the Installation and Administration manual (https://cran.r-project.org/doc/manuals/r-release/R-admin.html) provides instructions to install R on major plaforms (Windows, Linux, macOs). Once R has been installed, the requiered packages can be installed from the R command line using the following:

```
#Install BGLR package
install.packages("BGLR",repos="https://cran.r-project.org/")

#Install agricolae package
install.packages("agricolae",repos="https://cran.r-project.org/")

#Install doBy package
install.packages("doBy",repos="https://cran.r-project.org/")

#Install gplots package
install.packages("gplots",repos="https://cran.r-project.org/")
```

## Sample analysis

The documents **model\_fitting.html**, **FW.html** and **Biplot.html** contain scripts that illustrate how to perform the analysis presented in the study. To run the code we provide “toy datasets”; because these data sets include a very small fraction of the data used in the study, the results are not expected to match those presented in the study. The sole purpose of providing these ‘sample data sets’ is to demonstrate how the methods can be implemented.
