# Supplementary material for: A data-driven simulation platform to predict cultivars’ performances under uncertain weather conditions
Source: Nat Commun. 2020 Sep 25;11:4876. doi: 10.1038/s41467-020-18480-y (PMC7519145; doi:10.1038/s41467-020-18480-y)
Supplement: Supplementary file 2 — Supplementary Software [file 41467_2020_18480_MOESM2_ESM.zip › CODE/FW.html]

Finlay-Wilkinson


# Finlay-Wilkinson

#### G. de los Campos and P. Perez-Rodriguez

#### 03/23/2020

## Scripts for Finlay-Wilkinson analysis

Here we present a function to perform the Finlay-Wilkinson (1963) analysis in two steps using the BGLR (Pérez and de los Campos) package in R (R Core Team, 2019). We created the R function FW.BGLR to perform the analysis. The function takes the following arguments:

```
- pheno: a data.frame with 3 columns, VAR (variety), 
  ENV (environment), y (response variable).
- X: matrix of markers coded for additive effects (e.g, 0, 1, 2).
- G: matrix with genomic relationships between individuals.
```

The matrix **X** contains marker information for the varieties given in the data.frame. This matrix is used to compute the additive relationships between individuals (Lopez-Cruz et al., 2015) if matrix **G** is not given.

The function returns a list object with the following elements:

```
- yHat: vector with predicted values for the response variable.
- VAR: a data frame with the columns ID (varieties), 
  int (estimated intercept), intSD (estimated standard deviation for intercept),
  slope (estimated slope) and  slopeSD (estimated standard deviation for slope).
- ENV: vector with environmental effects.
```

The following R code shows how to load sample data (raw means).

```
#Set working directory 
setwd("~/Dropbox/ARVALIS_SIMULATION/SUBMISSION/SECOND_REVISION/FINAL_DOCUMENTS/Scripts")

#Load data
load("sample_FW.RData")

#list objects, at this point you should have at least 2 objects: pheno and G.
ls()
```

```
## [1] "G"     "pheno"
```

The code below shows how to load the function and perform the analysis assuming that objects pheno and G are already loaded in the R environment.

```
#Loads BGLR library
library(BGLR)

#Loads function for FW analysis
source("FW.BGLR.R")

#Pheno and G are already loaded

fm<-FW.BGLR(pheno=pheno,G=G,verbose=FALSE)

#Extracting results

#Predictions
head(fm$yHat)
```

```
##       [,1]
## 1 8.647097
## 2 9.044165
## 3 8.563319
## 4 9.365477
## 5 9.703875
## 6 9.350838
```

```
#Intercept and slopes
head(fm$VAR)
```

```
##    ID      int      intSD     slope    slopeSD
## G1 G1 9.317635 0.07183857 0.9549098 0.06229446
## G2 G2 9.117633 0.07458774 0.9841470 0.06066347
## G3 G3 9.381030 0.07866817 1.0984773 0.06291041
## G4 G4 9.453690 0.08660232 0.9671338 0.06669200
## G5 G5 9.669964 0.07432413 1.0539344 0.06483137
## G6 G6 9.441239 0.07363221 1.0531560 0.06068311
```

```
#Environmental effects
head(fm$ENV)
```

```
##       LOC1      LOC10      LOC11      LOC12      LOC13      LOC14 
## -0.3890250  1.6363510 -0.5031196 -1.8157042 -0.5608244 -1.9573635
```

```
#Plot Environment effect vs Genotype performance
plot.FW(fm,pheno)
```

### References

Finlay K.W. and G.N. Wilkinson. 1963. “The Analysis of Adaptation in a Plant-Breeding Programme.” Australian Journal of Agricultural Research 14 (6). CSIRO PUBLISHING:742. https://doi.org/10.1071/AR9630742.

Lopez-Cruz M., J. Crossa, D. Bonnett, S. Dreisigacker, J. Poland, J.L. Jannink, R.P. Singh, E. Autrique, and G. de los Campos. 2015. “Increased Prediction Accuracy in Wheat Breeding Trials Using a Marker x Environment Interaction Genomic Selection Model.” G3 (Bethesda, Md.) 5 (4). G3: Genes, Genomes, Genetics:569–82. https://doi.org/10.1534/g3.114.016097.

Pérez, P. and G. de los Campos. 2014. Genome-Wide Regression and Prediction with the BGLR Statistical Package. Genetics, 198: 483-495.

R Core Team. 2019. R: A language and environment for statistical computing. R Foundation for Statistical Computing, Vienna, Austria. URL https://www.R-project.org/.
