# Supplementary material for: A data-driven simulation platform to predict cultivars’ performances under uncertain weather conditions
Source: Nat Commun. 2020 Sep 25;11:4876. doi: 10.1038/s41467-020-18480-y (PMC7519145; doi:10.1038/s41467-020-18480-y)
Supplement: Supplementary file 2 — Supplementary Software [file 41467_2020_18480_MOESM2_ESM.zip › CODE/Biplot.html]

Biplot


# Biplot

#### G. de los Campos and P. Perez-Rodriguez

#### 03/23/2020

## Scripts Biplot analyses

We analyzed the simulated phenotypes using the site-regression model (SREG, Frutos et al., 2014) that is \(\tilde y\_{ij}=\mu+loc\_j + \sum\_{k=1}^t \lambda\_k \alpha\_{ik} \gamma\_{jk} + e\_{ij}\) where \(\tilde y\_{ij}\) is the average (simulated) grain yield of cultivar \(i\) in location \(j\), \(loc\_j\) is the main effect of location \(j\) and \(\lambda\_k \alpha\_{ik} \gamma\_{jk}\) is the \(k\)-th interaction component, corresponding to the \(ij\)-th element of singular value decomposition of the matrix of interactions. We estimated \(\mu\) and \(loc\_j\) using the least squares estimators, that is \(\hat \mu=\bar{\tilde{y}}..\) (the general mean), \(\hat{loc\_j}=\bar{\tilde{y}}.\_j\) (the mean of a site). We then computed \(z\_{ij}=\tilde y\_{ij}-\hat \mu - \hat{loc\_j}\), which can be arranged in matrix **Z** with cultivars in rows and locations in columns, and computed the Singular Value Decomposition (SVD) of **Z** to produce biplots.

The code below uses the data in `sample_simulated_yld.RData`, which contains a data frame with the average simulated yield for a subset of the genoypes in a subset of the locations.

### Loading the data

```
#Set working directory 
setwd("~/Dropbox/ARVALIS_SIMULATION/SUBMISSION/SECOND_REVISION/FINAL_DOCUMENTS/Scripts")

#Load sample data
load("sample_simulated_yld.RData")
```

### Biplot

```
#Load libraries
library(gplots)
```

```
## 
## Attaching package: 'gplots'
```

```
## The following object is masked from 'package:stats':
## 
##     lowess
```

```
locations<-levels(avg_yld$location)
varieties<-levels(avg_yld$variety)

#Create a matrix with as many rows as varieties and as many columns as 
#locations

Y<-matrix(NA,nrow=length(varieties),ncol=length(locations))

for(j in 1:length(locations))
{
  tmp<-avg_yld[avg_yld$location==locations[j],]
    Y[,j]<-tmp$yavg
}

colnames(Y)<-locations
rownames(Y)<-varieties

#Generate heatmap

heatmap.2(Y,cexRow=0.70,cexCol=0.6,trace="none",scale="none",
          srtCol=30)
```

```
#Biplots

#SREG, step by step computations
#Mean by site
Means<-colMeans(Y)

Z<-matrix(NA,nrow=nrow(Y),ncol=ncol(Y))

for(j in 1:ncol(Y))
{
    Z[,j]<-Y[,j]-Means[j]
}

rownames(Z)<-rownames(Y)

#Compute Singular value decomposition
out_svd<-svd(Z)

U<-out_svd$u
V<-out_svd$v

#Proportion of variance explained
proportions<-out_svd$d^2/sum(out_svd$d^2)*100

genotypes<-rownames(Z)
environments<-levels(avg_yld$location)

#Plot PC1 vs PC2
plot(U[,1:2],pch=19,xlim=c(-0.65,0.65),ylim=c(-0.55,0.55),
     xlab="PC1 (93.79 %)", ylab="PC2(3.72%)")

#Add the genotype labels
set.seed(7)
for(i in 1:nrow(U))
{
    text(x=U[i,1],y=U[i,2],
         labels=genotypes[i],pos=sample(c(1,2,3,4),1),cex=0.5)
}

#Add the arrows and the labels for locations
set.seed(1)
for(i in 1:nrow(V))
{

    arrows(0,0,V[i,1],V[i,2],length=0.10,col="blue")
    text(x=V[i,1],y=V[i,2],
         labels=colnames(Y)[i],pos=sample(c(1,2,3,4),1),
         cex=0.5,col="red")
}
```

### References

Frutos E., M. Purificación Galindo, and V. Leiva. 2014. “An Interactive Biplot Implementation in R for Modeling Genotype-by-Environment Interaction.” Stochastic Environmental Research and Risk Assessment 28 (7):1629–41. https://doi.org/10.1007/s00477-013-0821-z.

R Core Team. 2019. R: A language and environment for statistical computing. R Foundation for Statistical Computing, Vienna, Austria. URL https://www.R-project.org/.
