# Supplementary material for: A data-driven simulation platform to predict cultivars’ performances under uncertain weather conditions
Source: Nat Commun. 2020 Sep 25;11:4876. doi: 10.1038/s41467-020-18480-y (PMC7519145; doi:10.1038/s41467-020-18480-y)
Supplement: Supplementary file 2 — Supplementary Software [file 41467_2020_18480_MOESM2_ESM.zip › CODE/Model_fitting.html]

Model fitting


# Model fitting

#### G. de los Campos & P. Perez-Rodriguez

#### 03/23/2020

## Scripts Used to Fit the Models Using BGLR

This section contains supplementary R code for fitting the models used in the study. All the models were fitted using the BGLR package (Pérez and de los Campos, 2014) in R (R Core Team, 2019). The R software can be downloaded from R-website, and the BGLR package from CRAN-website or from the Github repository for BGLR.

Here, we present a prototype of the code needed to fit each of the models. We assume that the the data are loaded in into R environment including:

```
- pheno, a data frame with 5 columns:
  - $yield: (nx1), a numeric vector with the response variable.
  - $year: (nx1) a factor giving the IDs for the years. 
  - $location: (nx1), a factor giving the IDs for locations.
  - $yloc: (nx1), a factor with year-location combinations. 
  - $var: (nx1), a factor giving the IDs for the cultivars.
- G: a (qxq, q=number of cultivares) genomic relationship matrix (rownames are cultivar IDs).
- W: a (nxp) of environmental covariates.
```

The models are fitted using the BGLR function in R. In order to fit the models it is necessary to build the linear predictor (ETA), see, see Pérez and de los Campos (2014) or the examples in the Github repository for BGLR for more details and examples.

We include a sample dataset (“sample\_pheno\_geno\_W.RData”) that can be used to illustrate how to fit the models discussed. The following fragment of code shows how to load the data.

**Note:** The example below are only provided to show how models can be fitted. The results based on the sample data set, which includes a very small fraction of the data used in the study, may not match the ones in the publication.

### Loading data

```
#Set working directory 
setwd("~/Dropbox/ARVALIS_SIMULATION/SUBMISSION/SECOND_REVISION/FINAL_DOCUMENTS/Scripts")

#Load data
load("sample_pheno_geno_W.RData")

#BGLR
library(BGLR)
#list objects, at this point you should have at least 3 objects: pheno, G and W.
ls()
```

```
## [1] "G"     "pheno" "W"
```

### Baseline model

```
nIter<-12000 #set only for the purpouse of the example, in practice more may be needed
burnIn<-2000
thin<-10

set.seed(195021) 
#Incidence matrix for main effect of years
pheno$year<-as.factor(pheno$year)
ZY<-model.matrix(~pheno$year-1)

#Incidence matrix for main effect of location
pheno$location<-as.factor(pheno$location)
ZL<-model.matrix(~pheno$location-1)

#Incidence matrix for main effect of year-location
pheno$yloc <-as.factor(pheno$yloc)
ZYL<-model.matrix(~pheno$yloc-1)

#Incidence matrix for main eff. of lines.
pheno$var<-factor(x=pheno$var,levels=rownames(G),ordered=TRUE)
Zv<-model.matrix(~pheno$var-1)

#Linear predictor(ETA)
Eta1<-list(year=list(X=ZY,model='BRR',saveEffects=TRUE ),
           loc=list(X=ZL,model='BRR',saveEffects=TRUE),
           yloc=list(X=ZYL,model='BRR',saveEffects=TRUE),
           v=list(X=Zv,model='BRR',saveEffects=TRUE))

#Fitting the model
fm1<-BGLR(y=pheno$y,ETA=Eta1,nIter=nIter,burnIn=burnIn,thin=thin,saveAt='m1_',
          verbose=FALSE)

#Predictions can be retrieved using
  # - Year: fm1$ETA$year$b
  # - Locations: fm1$ETA$loc$b
  # - Year-loc: fm1$ETA$yloc$b
  # - Gentoypes: fm1$v$b
```

**Variance Components:**

```
 par(mfrow=c(2,3))

 varE<-scan("m1_varE.dat")
 
 #Trace plot
 plot(varE,type="b",xlab="Thinned iteration",ylab="varE")
 
 mean(varE)
```

```
## [1] 0.3932613
```

```
 #Year-variance
 B<-readBinMat('m1_ETA_year_b.bin') # posterior samples
 TMP<-tcrossprod(ZY,B)
 vY<-apply(FUN=var,X=TMP,MARGIN=2)
 
 #Trace plot
 plot(vY,type="b",
      xlab="Thinned iteration",ylab="Year variance") 
 
 #Location
 B<-readBinMat('m1_ETA_loc_b.bin') # posterior samples 
 TMP<-tcrossprod(ZL,B)
 vL<-apply(FUN=var,X=TMP,MARGIN=2)
 
 #Trace plot
 plot(vL,type="b",
      xlab="Thinned iteration",ylab="Loc. variance") 
 
 #Year-Location
 B<-readBinMat('m1_ETA_yloc_b.bin') # posterior samples
 TMP<-tcrossprod(ZYL,B)
 vYL<-apply(FUN=var,X=TMP,MARGIN=2)

 #Trace plot
 plot(vYL,type="b",
      xlab="Thinned iteration",ylab="Year-Loc variance") 
 
 #Cultivar
 B<-readBinMat('m1_ETA_v_b.bin') # posterior samples
 TMP<-tcrossprod(Zv,B)
 vG<-apply(FUN=var,X=TMP,MARGIN=2)
 #Trace plot
 plot(vG,type="b",
      xlab="Thinned iteration",ylab="Cultivar variance")
```

### GW model

Note: in the implementations presented below, we modeled genetic effects using the PCs of the genomic relationship matrix. Other alternatives includes using the Cholesky decomposition or the G-matrix directly. For examples please look at the following entry.

```
EVD.G<-eigen(G)
EVD.G$vectors<-EVD.G$vectors[,EVD.G$values>1e-8]
EVD.G$values<-EVD.G$values[EVD.G$values>1e-8]

#Center and standardize environmental covariates
W<-scale(W,center=TRUE,scale=TRUE)
W<-W/sqrt(ncol(W))

PC.G<-sweep(EVD.G$vectors,MARGIN=2,STATS=sqrt(EVD.G$values),FUN='*')
ZPC.G<-Zv%*%PC.G

#Linear predictor(ETA)
Eta2<-list(v=list(X=ZPC.G,model='BRR',saveEffects=TRUE),
           ec=list(X=W,model='BRR',saveEffects=TRUE))

#Fitting the model
fm2<-BGLR(y=pheno$y,ETA=Eta2,nIter=nIter,burnIn=burnIn,thin=thin,saveAt='m2_',
          verbose=FALSE)

#BLUPS for genotypes?
 #- uHat<-ZPC.G%*%fm2$ETA$v$b

#Variance components
 par(mfrow=c(2,2))
 
 #Use this vector to compute posterior mean, 
 #posterior SD, or credibility regions
 
 plot(scan('m2_varE.dat'),type="b",
      xlab="Thinned iteration",ylab="varE") 
 
 #Env Cov
 B<-readBinMat('m2_ETA_ec_b.bin') # posterior samples
 TMP<-tcrossprod(W,B)
 vEC<-apply(FUN=var,X=TMP,MARGIN=2)
 #Trace plot
 plot(vEC,xlab="Thinned iteration",ylab="EC variance") 
 
 #Cultivars
 B<-readBinMat('m2_ETA_v_b.bin') # posterior samples 
 TMP<-tcrossprod(ZPC.G,B)
 vL<-apply(FUN=var,X=TMP,MARGIN=2) 
 #trace plot
 plot(vL,type="b",xlab="Thinned iteration",
      ylab="Cultivar variance")
```

### GW-GxW model

```
Omega<-tcrossprod(W)
GxW<-tcrossprod(ZPC.G)*Omega

EVD.GW<-eigen(GxW)
EVD.GW$vectors<-EVD.GW$vectors[,EVD.GW$values>1e-8]
EVD.GW$values<-EVD.GW$values[EVD.GW$values>1e-8]

PC.GW<-sweep(EVD.GW$vectors,MARGIN=2,STATS=sqrt(EVD.GW$values),FUN='*')

  
#Linear predictor(ETA)
Eta3<-list(v=list(X=ZPC.G,model='BRR',saveEffects=TRUE),
           ec=list(X=W,model='BRR',saveEffects=TRUE),
           GxW=list(X=PC.GW,model='BRR',saveEffects=TRUE)) 

#Fitting the model
fm3<-BGLR(y=pheno$y,ETA=Eta3,nIter=nIter,burnIn=burnIn,thin=thin,saveAt='m3_',
          verbose=FALSE)

# Pediction of GW effects?   PC.GW%*%fm3$ETA[['GxW']]$b

# Variance components
 par(mfrow=c(2,2))
 varE<-scan("m3_varE.dat")
 
 #Trace plot
 plot(varE,type="b",xlab="Thinned iteration",
      ylab="varE")
 mean(varE)
```

```
## [1] 1.005483
```

```
 #Cultivar
 B<-readBinMat('m3_ETA_v_b.bin') # posterior samples
 TMP<-tcrossprod(ZPC.G,B)
 vG<-apply(FUN=var,X=TMP,MARGIN=2)
 #Trace plot
 plot(vG,type="b",xlab="Thinned iteration",
      ylab="Cultivar variance")  

 #Environmental convariates
 B<-readBinMat('m3_ETA_ec_b.bin') # posterior samples
 TMP<-tcrossprod(W,B)
 vEC<-apply(FUN=var,X=TMP,MARGIN=2)
 #Trace plot
 plot(vEC,type="b",xlab="Thinned iteration",ylab="EC variance")  

 
 #GxW
 B<-readBinMat('m3_ETA_GxW_b.bin') # posterior samples
 TMP<-tcrossprod(PC.GW,B)
 vGW<-apply(FUN=var,X=TMP,MARGIN=2)
 #Trace plot
 plot(vGW,type="b",xlab="Thinned iteration",
      ylab="SNPxEC variance")
```

### TGW model

```
#Linear predictor(ETA)
Eta4<-list(year=list(X=ZY,model='BRR',saveEffects=TRUE),
           loc=list(X=ZL,model='BRR',saveEffects=TRUE),
           yloc=list(X=ZYL,model='BRR',saveEffects=TRUE),
           v=list(X=ZPC.G,model='BRR',saveEffects=TRUE),
           ec=list(X=W,model='BRR',saveEffects=TRUE))

#Fitting the model
fm4<-BGLR(y=pheno$y,ETA=Eta4,nIter=nIter,burnIn=burnIn,thin=thin,saveAt='m4_',
          verbose=FALSE)

# Variance components
 par(mfrow=c(3,2))
 varE<-scan("m4_varE.dat")
 #Trace plot
 plot(varE,type="b",xlab="Thinned iteration",ylab="varE")
 mean(varE)
```

```
## [1] 0.3910069
```

```
 # Year-variance
 B<-readBinMat('m4_ETA_year_b.bin') # posterior samples
 TMP<-tcrossprod(ZY,B)
 vY<-apply(FUN=var,X=TMP,MARGIN=2)
 #Trace plot
 plot(vY,type="b",xlab="Thinned iteration",ylab="Year variance") 
 
 #Location
 B<-readBinMat('m4_ETA_loc_b.bin') # posterior samples 
 TMP<-tcrossprod(ZL,B)
 vL<-apply(FUN=var,X=TMP,MARGIN=2)
 #trace plot
 plot(vL,type="b",xlab="Thinned iteration",ylab="Loc. variance") 
 
 #Year-Location
 B<-readBinMat('m4_ETA_yloc_b.bin') # posterior samples
 TMP<-tcrossprod(ZYL,B)
 vYL<-apply(FUN=var,X=TMP,MARGIN=2)
 #Trace plot
 plot(vYL,xlab="Thinned iteration",ylab="Year-Loc. variance") 
 
 #Cultivar
 B<-readBinMat('m4_ETA_v_b.bin') # posterior samples
 TMP<-tcrossprod(ZPC.G,B)
 vG<-apply(FUN=var,X=TMP,MARGIN=2)
 #Trace plot
 plot(vG,type="b",xlab="Thinned iteration",ylab="Cultivar variance")  

 #EC
 B<-readBinMat('m4_ETA_ec_b.bin') # posterior samples
 TMP<-tcrossprod(W,B)
 vEC<-apply(FUN=var,X=TMP,MARGIN=2)
 #Trace plot
 plot(vEC,type="b",xlab="Thinned iteration",ylab="EC variance")
```

### TGW-GxW

```
#Linear predictor(ETA)
Eta5<-list(year=list(X=ZY,model='BRR',saveEffects=TRUE),
           loc=list(X=ZL,model='BRR',saveEffects=TRUE),
           yloc=list(X=ZYL,model='BRR',saveEffects=TRUE),
           v=list(X=ZPC.G,model='BRR',saveEffects=TRUE),
           ec=list(X=W,model='BRR',saveEffects=TRUE),
           GxW=list(X=PC.GW,model='BRR',saveEffects=TRUE))

#Fitting the model
fm5<-BGLR(y=pheno$y,ETA=Eta5,nIter=nIter,burnIn=burnIn,thin=thin,saveAt='m5_',
          verbose=FALSE)

#Variance components
 par(mfrow=c(3,3))
 varE<-scan("m5_varE.dat")
 #Trace plot
 plot(varE,type="b",xlab="Thinned iteration",ylab="varE")
 mean(varE)
```

```
## [1] 0.3657147
```

```
 #Year-variance
 B<-readBinMat('m5_ETA_year_b.bin') # posterior samples
 TMP<-tcrossprod(ZY,B)
 vY<-apply(FUN=var,X=TMP,MARGIN=2)
 #Trace plot
 plot(vY,type="b",xlab="Thinned iteration",ylab="Year variance") 
 
 # Location
 B<-readBinMat('m5_ETA_loc_b.bin') # posterior samples 
 TMP<-tcrossprod(ZL,B)
 vL<-apply(FUN=var,X=TMP,MARGIN=2)
 
 #Trace plot
 plot(vL,type="b",xlab="Thinned iteration",ylab="Loc. variance") 
 
 #Year-Location
 B<-readBinMat('m5_ETA_yloc_b.bin') # posterior samples
 TMP<-tcrossprod(ZYL,B)
 vYL<-apply(FUN=var,X=TMP,MARGIN=2)
 #Trace plot
 plot(vYL,type="b",xlab="Thinned iteration",ylab="Year-Loc variance") 
 
 #Cultivar
 B<-readBinMat('m5_ETA_v_b.bin') # posterior samples
 TMP<-tcrossprod(ZPC.G,B)
 vG<-apply(FUN=var,X=TMP,MARGIN=2)
 #Trace plot
 plot(vG,type="b",xlab="Thinned iteration",ylab="Cultivar variance")  

 #EC
 B<-readBinMat('m5_ETA_ec_b.bin') # posterior samples
 TMP<-tcrossprod(W,B)
 vEC<-apply(FUN=var,X=TMP,MARGIN=2)
 #Trace plot
 plot(vEC,type="b",xlab="Thinned iteration",ylab="EC variance")  

 #GW
 B<-readBinMat('m5_ETA_GxW_b.bin') # posterior samples
 TMP<-tcrossprod(PC.GW,B)
 vGW<-apply(FUN=var,X=TMP,MARGIN=2)
 #Trace plot
 plot(vGW,type="b",xlab="Thinned iteration",ylab="SNPxEC variance")
```

### Template for evaluating prediction accuracy

To evaluate prediction accuracy one can set the entries of the phenotype vector corresponding to the testing set to `NA` and predictions for these entries can be retrieved from the fitted model. The example below assigns NAs at random and evaluates predictions across year locations. In the study we conducted 2 CVs, for details we refer to the Materials and Methods section.

```
yNA<-pheno$y
tst<-sample(1:length(yNA),size=100)
yNA[tst]<-NA

#Fitting the model

fm4NA<-BGLR(y=pheno$y,ETA=Eta4,nIter=nIter,burnIn=burnIn,thin=thin,
            saveAt='tst_',verbose=FALSE)

# note this correlation is substantially higher than what we report 
# because this correlation is across year-locations and NAs 
# were generated at random.

cor(pheno$y[tst],fm4NA$yHat[tst])
```

```
## [1] 0.9186689
```

### References

Pérez, P. and G. de los Campos. 2014. Genome-Wide Regression and Prediction with the BGLR Statistical Package. Genetics, 198: 483-495.

R Core Team. 2019. R: A language and environment for statistical computing. R Foundation for Statistical Computing, Vienna, Austria. URL https://www.R-project.org/.
