# Supplementary material for: A data-driven simulation platform to predict cultivars’ performances under uncertain weather conditions
Source: Nat Commun. 2020 Sep 25;11:4876. doi: 10.1038/s41467-020-18480-y (PMC7519145; doi:10.1038/s41467-020-18480-y)
Supplement: Supplementary file 4 — Reporting Summary [file 41467_2020_18480_MOESM4_ESM.pdf]

nature research

Corresponding author(s): Gustavo de los Campos, Paulino Perez-Rodriguez, Matthieu Bogard

Last updated by author(s): 8/12/2020

Reporting Summary

Nature Research wishes to improve the reproducibility of the work that we publish. This form provides structure for consistency and transparency in reporting. For further information on Nature Research policies, see our Editorial Policies and the Editorial Policy Checklist.

Statistics

For all statistical analyses, confirm that the following items are present in the figure legend, table legend, main text, or Methods section.

n/a	Confirmed	
☐	☒	The exact sample size (*n*) for each experimental group/condition, given as a discrete number and unit of measurement
☐	☒	A statement on whether measurements were taken from distinct samples or whether the same sample was measured repeatedly
☐	☒	The statistical test(s) used AND whether they are one- or two-sided<br>*Only common tests should be described solely by name; describe more complex techniques in the Methods section.*
☐	☒	A description of all covariates tested
☐	☒	A description of any assumptions or corrections, such as tests of normality and adjustment for multiple comparisons
☐	☒	A full description of the statistical parameters including central tendency (e.g. means) or other basic estimates (e.g. regression coefficient) AND variation (e.g. standard deviation) or associated estimates of uncertainty (e.g. confidence intervals)
☐	☒	For null hypothesis testing, the test statistic (e.g. *F*, *t*, *r*) with confidence intervals, effect sizes, degrees of freedom and *P* value noted<br>*Give P values as exact values whenever suitable.*
☐	☒	For Bayesian analysis, information on the choice of priors and Markov chain Monte Carlo settings
☐	☒	For hierarchical and complex designs, identification of the appropriate level for tests and full reporting of outcomes
☐	☒	Estimates of effect sizes (e.g. Cohen's *d*, Pearson's *r*), indicating how they were calculated

Our web collection on statistics for biologists contains articles on many of the points above.

Software and code

Policy information about availability of computer code

Data collection	No specific software was used for field data collection.
Data analysis	All analyses were done using functions including in R and R-packages (BGLR, agricolae), plus custom scripts. Scripts are provided as supplementary materials

For manuscripts utilizing custom algorithms or software that are central to the research but not yet described in published literature, software must be made available to editors and reviewers. We strongly encourage code deposition in a community repository (e.g. GitHub). See the Nature Research guidelines for submitting code & software for further information.

Data

Policy information about availability of data

All manuscripts must include a data availability statement. This statement should provide the following information, where applicable:

- Accession codes, unique identifiers, or web links for publicly available datasets
- A list of figures that have associated raw data
- A description of any restrictions on data availability

The data that support the findings of this study are available from Arvalis but restrictions apply to the availability of these data, which were used under license for the current study, and so are not publicly available. Data are however available from the authors upon reasonable request and with permission of Arvalis.

Field-specific reporting

Please select the one below that is the best fit for your research. If you are not sure, read the appropriate sections before making your selection.

☒ Life sciences ☐ Behavioural & social sciences ☐ Ecological, evolutionary & environmental sciences

For a reference copy of the document with all sections, see nature.com/documents/nr-reporting-summary-flat.pdf

Life sciences study design

All studies must disclose on these points even when the disclosure is negative.

Sample size	Sample size was determined by the data collected by Arvalis in their network of trials.
Data exclusions	None
Replication	We conducted several forms of cross-validation.
Randomization	The assignment of genotypes to trials and plots within year was done at random.
Blinding	No treatment effects were tested in this study.

Reporting for specific materials, systems and methods

We require information from authors about some types of materials, experimental systems and methods used in many studies. Here, indicate whether each material, system or method listed is relevant to your study. If you are not sure if a list item applies to your research, read the appropriate section before selecting a response.

Materials & experimental systems

n/a	Involved in the study	
☒	☐	Antibodies
☒	☐	Eukaryotic cell lines
☒	☐	Palaeontology and archaeology
☐	☒	Animals and other organisms
☒	☐	Human research participants
☒	☐	Clinical data
☒	☐	Dual use research of concern

Methods

n/a	Involved in the study	
☒	☐	ChIP-seq
☒	☐	Flow cytometry
☒	☐	MRI-based neuroimaging

Animals and other organisms

Policy information about studies involving animals; ARRIVE guidelines recommended for reporting animal research

Laboratory animals	Triticum
Wild animals	NA
Field-collected samples	Phenotype data originated in post-registration evaluation trials carried out each year by Arvalis for newly registered varieties. The trial network included 242 locations and a total of 752 year-locations. Each year-location had one (un-replicated) yield trial in which cultivars were arranged in a randomized complete block design with a plot size of 12 m^2. Sowing dates and densities were adjusted at each location to represent the usual practices at each location. Trials were managed to reduce any biotic or abiotic factor that may reduce grain yield potential (optimal nitrogen fertilization, weed, insect, and disease control). Plots were harvested at maturity using a combine harvester, and the grain yield of each plot was adjusted to 15% moisture.
Ethics oversight	NA since no human or animal data was involved.

Note that full information on the approval of the study protocol must also be provided in the manuscript.
